# Capturing trophectoderm-like stem cells enables step-wisely remodeling of placental development

**DOI:** 10.1093/procel/pwaf098

**Published:** 2025-11-10

**Authors:** Xinyi Jia, Bing Peng, Hongjin Zhao, Chunhui Wang, Aibin He, Wei Tao, Peng Du

**Affiliations:** MOE Key Laboratory of Cell Proliferation and Differentiation, School of Life Sciences, Peking University, Beijing 100871, China; MOE Key Laboratory of Cell Proliferation and Differentiation, School of Life Sciences, Peking University, Beijing 100871, China; Shandong Provincial Hospital Affiliated to Shandong First Medical University, Jinan 250021, China; MOE Key Laboratory of Cell Proliferation and Differentiation, School of Life Sciences, Peking University, Beijing 100871, China; Institute of Molecular Medicine and National Biomedical Imaging Center, College of Future Technology, Peking-Tsinghua Center for Life Sciences and State Key Laboratory of Gene Function and Modulation Research, Peking University, Beijing 100871, China; MOE Key Laboratory of Cell Proliferation and Differentiation, School of Life Sciences, Peking University, Beijing 100871, China; MOE Key Laboratory of Cell Proliferation and Differentiation, School of Life Sciences, Peking University, Beijing 100871, China; Peking-Tsinghua Center for Life Sciences, Academy for Advanced Interdisciplinary Studies, Peking University, Beijing 100871, China; Beijing Advanced Center of RNA Biology, Peking University, Beijing 100871, China

**Keywords:** embryonic development, totipotency, stem cell, trophectoderm, trophoblast, placenta, organoid, epigenetic, transcriptomic, pregnancy

## Abstract

The trophectoderm produced from totipotent blastomeres initiates trophoblast development, while placental deficiencies can cause pregnancy disorders. Yet, a culture system that fully recapitulates the entire placenta development is still lacking, greatly limiting related studies. Here, we captured mouse trophectoderm-like stem cells (TELSCs), which can give rise to all trophoblast lineages and can be applied to generate trophoblast organoids. We achieved the induction and maintenance of TELSCs from totipotent blastomere-like stem cells or early embryos through a Hippo-YAP/Notch-to-TGFβ1 signaling switch. At the molecular level, TELSCs resemble E4.5 trophectoderm and are distinct from all previously known trophoblast-like stem cells. Functionally, TELSCs can generate all trophoblast lineages in both teratoma and chimera assays. We further applied TELSCs to generate trophoblast organoids containing various mature trophoblasts and a self-renewing extraembryonic ectoderm (ExE)-like progenitor population. Interestingly, we observed transiently formed rosette-like structures that rely on *Itgb1*, which are essential to induce ExE-like progenitors and to generate organoids eventually. Thus, the capture of TELSCs enables comprehensive insights into placental development.

## Introduction

During murine embryonic development, trophoblasts originate from the outer cells of compacted embryos at the eight-cell stage (Goolam et al., 2016; Johnson and Ziomek, 1981; Lim et al., 2020; Wang et al., 2018b; White et al., 2016; Zernicka-Goetz et al., 2009). By embryonic day 3.5 (E3.5), the trophectoderm (TE) and inner cell mass (ICM) specification has been completed (Chazaud and Yamanaka, 2016; Chen et al., 2018; Mihajlović and Bruce, 2017; Zhu and Zernicka-Goetz, 2020), culminating in embryo implantation into the uterus through the mural TE around E4.5 (Aplin and Ruane, 2017; Christodoulou et al., 2019). The mural TE undergoes differentiation into trophoblast giant cells (TGCs), while the polar TE proliferates, giving rise to the extraembryonic ectoderm (ExE) and ectoplacental cone (EPC), consisting of TGCs and spongiotrophoblasts (SpTs) (Christodoulou et al., 2019; Rossant and Cross, 2001; Senner and Hemberger, 2010; Simmons et al., 2008). Around E8.5, the chorionic epithelium derived from the ExE contacts the fetal mesoderm and differentiates into two layers of syncytiotrophoblasts (SynTs). Subsequently, the placental vascular network develops, forming the labyrinth, a densely packed structure (Latos and Hemberger, 2016; Shahbazi and Zernicka-Goetz, 2018; Ueno et al., 2013). The mature placenta, around E12.5, comprises outer TGCs, SpTs, and inner labyrinth trophoblasts, serving as a transient essential organ facilitating efficient fetal-maternal communication for successful embryo development and pregnancy. Given the prevalence of developmental disorders that cause pregnancy failure in humans (Aplin et al., 2020), comprehending the developmental program of trophoblast lineages and the placenta is crucial for reproductive and regenerative medicine.

*In vitro*, both human and mouse trophoblast stem cells (TSCs) have been derived from TE cells of blastocysts or ExE of post-implantation embryos (Tanaka et al., 1998), while under long-term culturing under defined conditions such as in TSC or TX medium, these mouse TSCs closely resemble trophoblast progenitors of E5.5–7.5 embryos (Kubaczka et al., 2014). Notably, recent studies have shown that naïve, or even primed, human iPSCs or embryonic stem cells (ESCs) can be induced to generate TSCs (Dong et al., 2020; Wei et al., 2021), while in contrast, mouse pluripotent ESCs cannot be induced to form TSCs, suggesting that human pluripotent iPSCs/ESCs possess much higher plasticity than mouse ESCs. Particularly, quite recently, a novel type of so-called trophectoderm stem cells (TESCs) has been captured in a newly developed medium supplemented with Activin, IL11, BMP7, 8-Br cAMP, and LPA, since transcriptomic comparison suggested that these cells may possess certain features of polar TE cells (Seong et al., 2022). However, a detailed comparison between TESCs and *in vivo* TE at different developmental stages is still required to clearly classify the status of TESCs.

However, the lack of an experimental model that accurately simulates *in vivo* conditions has limited our knowledge of placental development. Recently, human trophoblast organoids (TOs) derived from placental villous tissue and TSCs, either directly isolated *in vivo* or differentiated from human ESCs *in vitro*, have been established as tools for *in vitro* studies of placental development and disease (Haider et al., 2018; Karvas et al., 2022; Turco et al., 2018). However, these models exhibit an inside-out villous architecture and contain limited cell types, differing from native placental villi. Similarly, a recent study has demonstrated that mouse TOs can also be derived from placentas or TSCs (Mao et al., 2023). However, two separate media are required to support organoid proliferation and differentiation, and the differentiation culture system lacks syncytiotrophoblast layer II (SynTII) cell types. Furthermore, both human and mouse TSC-to-TO formation systems only briefly recapitulate post-implantation placental development. The initiation of TE fate specification and the pre-implantation TE state transitions still cannot be recaptured *in vitro*. Thus, a trophoblast differentiation system capable of mimicking the entire stepwise placental development process initiated from totipotent stem cells remains unavailable.

In this study, we captured a novel type of trophectoderm-like stem cells (TELSCs), which can be applied to the stepwise remodeling of the entire placental development. Based on the Hippo-YAP/Notch-to-TGFβ1 signaling switch, we developed the “two-step” system, which enabled the robust induction and stable propagation of TELSCs from both *in vitro* cultured totipotent blastomere-like cells (TBLCs) (Shen et al., 2021) and directly from *in vivo* eight-cell stage mouse embryos. Molecularly, TELSCs closely resemble the TE cells of E4.5 blastocysts at both transcriptomic and epigenetic levels, and are clearly distinct from conventional TSCs and recently reported TESCs. Remarkably, in mouse teratoma and chimera assays, we demonstrated that TELSCs were able to successfully produce all the placental trophoblast lineages at the single-cell level. Furthermore, TELSCs can be applied to readily generate TOs with all mature trophoblasts and long-term passaging ability. Additionally, we identified a novel population of E5.5–6.5 ExE-like progenitor cells with a high cell proliferation rate, which enabled the coupled self-renewal and differentiation abilities of TELSC-derived organoids. Interestingly, during organoid formation, we observed a dynamic and transient morphological formation of rosette-like structures, relying on the key β1 signaling factor *Itgb1*, which was essential to induce ExE-like progenitors and eventually to generate organoids from TELSCs. This achievement not only deepens our understanding of stepwise trophoblast differentiation from totipotent stem cells, but also provides a robust *in vitro* system for comprehensively investigating crucial events governing placental development.

## Results

### Mouse TELSCs were induced and stably maintained from TBLCs and eight-cell blastomeres using a “two-step” culture system

The placenta, comprising diverse trophoblast lineages derived from TE, plays a pivotal role in mediating fetal-maternal communication during pregnancy, and placental deficiency is implicated in various human fertility disorders. However, the lack of *in vitro* trophoblast culture and differentiation systems, particularly for the pre-implantation stage, has greatly impeded our understanding of placental development and related diseases to date. Recently, a novel kind of mouse TBLCs closely resembling two-/four-cell blastomeres, which can produce various embryonic and extraembryonic lineages, including mature trophoblasts, has been captured and stably maintained *in vitro* (Peng et al., 2025; Shen et al., 2021). Consequently, TBLCs serve as ideal “seed cells” for establishing a trophoblast differentiation system originating from totipotent stem cells.

**Figure 1.**
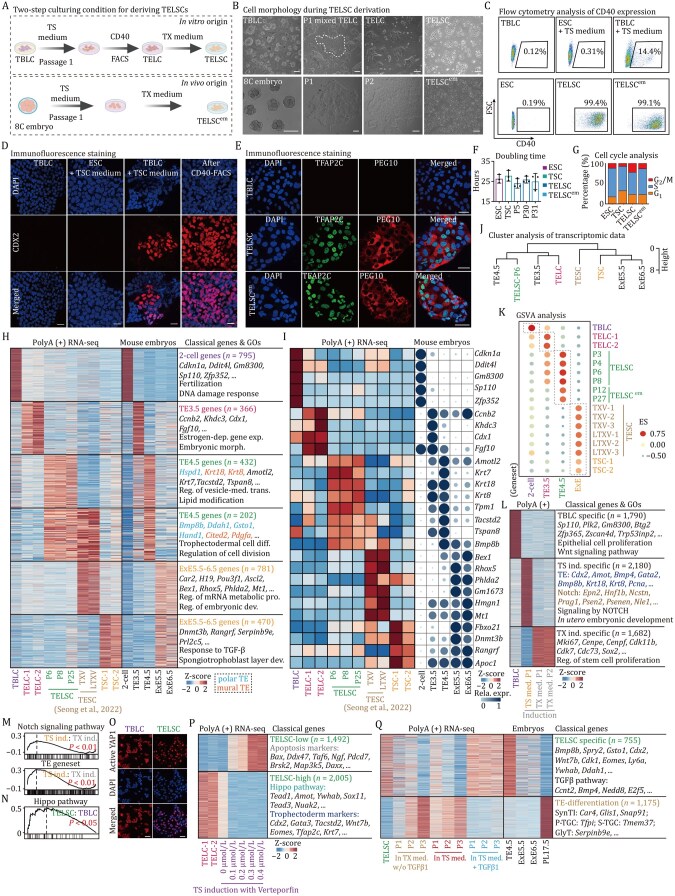
**Capturing trophectoderm-like stem cells (TELSCs) with pre-implantation E4. 5 TE features using a “two-step” culture system**. (A) The diagram illustrates the process to obtain TELSCs (upper panel) and TELSC^em^s (lower panel). (B) The morphology of TELSCs (upper panel) and TELSC^em^s at different stages (lower panel). Scale bars, 100 µm. (C) FACS analysis of the percentage of CD40^+^ cells from TBLCs, as well as ESCs and TBLCs cultured in TS medium, using the V6.5 cell line. (D) Immunofluorescence staining of CDX2 in CD40^+^ cells from ESCs and ESCs cultured in TS medium, as well as TBLCs cultured in TS medium before and after CD40 FACS purification. (E) Immunofluorescence staining of TFAP2C and PEG10 in TBLCs, TELSCs, and TELSC^em^s. Scale bars, 50 µm. (F) Cell doubling time of ESCs, TSCs, TELSCs, and TELSC^em^s at different passages. (G) Cell cycle analysis of ESCs, TSCs, TELSCs, and TELSC^em^s. (H) Heatmap indicating the relative expression of differentially expressed genes related to mouse embryos (Cheng et al., 2019; Sun et al., 2021; Wang et al., 2018a) in TBLCs, TELCs, TELSCs, TSCs, and trophectoderm stem cells (TESCs). Transcriptomic data of TESCs are from Seong et al. (2022). The enrichment of Gene Ontology (GO) terms of these genes is shown on the right. Dep., dependent; exp., expression; reg., regulation; trans., transport; diff.,differentiation; pro., process; dev., development. (I) Heatmap indicating the relative expression of characteristic genes in TBLCs, TELCs, TELSCs, TESCs, and TSCs. Bubble chart showing the relative expression of these genes in mouse embryos. (J) Hierarchical clustering analysis of TELCs, TELSCs, TSCs, TESCs, and mouse embryos data. (K) Gene set variation analysis (GSVA) analysis of TBLCs, TELCs, TELSCs, TELSC^em^s, TESCs, TSCs, and mouse embryos data. (L) Heatmap indicating the relative expression of differentially expressed genes of TBLCs and TBLCs induction in TS medium and TX medium. (M) Gene Set Enrichment Analysis (GSEA) analysis of TBLCs induction in TS medium and TX medium based on Notch signaling pathway and TE geneset. (N) GSEA of TELSCs versus TBLC samples based on Hippo pathway geneset. (O) Immunoﬂuorescence staining of active YAP1 in TELSCs and TBLCs. Scale bars, 50 µm. (P) Heatmap indicating the differentially expressed genes of TELCs and TBLCs induction in TS medium plus verteporfin. The representative genes and enrichment of GO terms of these genes are shown. (Q) Heatmap indicating the differentially expressed genes of TELSCs, TBLCs induction in TX medium, withdrawal of TGFβ1, in TS medium, and in TS medium plus TGFβ1. Heatmap on the right demonstrating the expression of each cluster in mouse embryos. The representative genes and enrichment of GO terms of these genes are shown.

We first plated ESCs and TBLCs into classical serum-containing TS medium (Tanaka et al., 1998) supplemented with various factors known to induce trophoblast lineages, such as FGF4 (Kubaczka et al., 2014), Activin A (Ohinata and ­Tsukiyama, 2014), TGFβ1 (Erlebacher et al., 2004), and BMP4 (Tsuchida et al., 2020). After three days, TBLCs exhibited significant morphological changes, forming tight epithelium-like colonies characteristic of trophoblast cells specifically in the FGF4-containing TS medium, but not in other media. Notably, ESCs did not respond similarly (Figs. 1A, 1B and S1A). Fluorescence-activated cell sorting (FACS) analysis on the typical TE-specific markers, including CDX2 and CD40, revealed a notable population of CDX2^+^/CD40^+^ TE-like cells, constituting approximately 14% of TBLC-derived cells. While pluripotent ESCs cultured under the same conditions failed to generate a similar cell population (Figs. 1C, S1B and S1C). Further immunostaining analysis confirmed the successful induction of TE-like cells from TBLCs at the protein level (Fig. 1D). Thus, TE-like cells can be easily and efficiently induced from TBLCs in TS medium, but not from pluripotent ESCs, and we name these transiently induced TE-like cells as trophectoderm-like cells (TELCs).

However, these TELCs could not sustain a homogeneous morphology or undergo long-term passaging in the serum-containing TS medium (Fig. S1F). Instead, they were stably maintained with stable TE-like morphology for more than 30 passages in the recently reported serum-free TX medium (Kubaczka et al., 2014) (Fig. 1A and 1B). Utilizing CD40-based FACS analysis, we observed that more than 99% of cells remained positive across various passages (Fig. 1C), suggesting their steady and homogeneous cell status, which can be further confirmed by Western blot and immunostaining analysis by employing antibodies against TE-specific markers, including CDX2, EOMES, TFAP2C, and PEG10 (Figs 1E, 1J and S1G). We named these cells stably maintained in TX medium as TELSCs.

Similarly, we plated embryos at different developmental stages in the same “two-step” culturing system and found that eight-cell embryos could give rise to homogeneous cells with TE morphology, which could be stably maintained over long-term passages (referred to as TELSC^em^; Figs. 1A, 1B and S1H). FACS analysis revealed that over 99% of these embryo-derived cells were CD40-positive (Figs. 1C and S1I), and immunostaining confirmed robust expression of key TE lineage markers, including CDX2, EOMES, HAND1, TFAP2C, and PEG10 (Figs. 1E and S1J). Notably, TELSCs derived from both TBLCs and eight-cell embryos display rapid self-renewal ability, with a much higher proliferation rate than conventional TSCs, even after 30 passages (Fig. 1F). While cell cycle analysis revealed that TELSCs maintained a typical active stem cell-like cycle distribution, characterized by an increased G_2_/M phase and a reduced S phase compared to pluripotent ESCs (Figs. 1G and S1K).

Above all, we developed a “two-step” TS-TX culture strategy that enables the efficient and reproducible derivation of a novel type of TELSCs with typical TE features and a fast self-renewal ability from mouse TBLCs or eight-cell embryos.

### TELSCs are distinct from known TESCs/TSCs and are close to pre-implantation E4.5 TE cells at the transcriptomic level

Subsequently, we aimed to comprehensively characterize the molecular attributes of TELSCs stably maintained in TX medium, as well as transiently induced TELCs in TS medium. RNA-seq was performed on these cells, and transcriptomic comparison clearly showed that compared to the original TBLCs, a total of 795 totipotent genes, including *Gm8300*, *Zfp352*, *Ddit4l*, and *Sp100*, were uniformly silenced in TELCs and TELSCs. While in TELCs, 366 genes represented by *Ccnb2*, *Cdx1*, and *Fgf10*, which are particularly enriched in TE cells of E3.5 embryos (TE3.5) and are related to “Estrogen-dependent gene expression,” were dynamically and specifically activated. Furthermore, a large group of genes, such as *Amotf2*, *Krt7*/*8*/*18*, *Tspan8*, *Pdgfa*, *Cited2*, *Hand1*, and *Tacstd2*, which are preferentially enriched in TE cells of E4.5 embryos (TE4.5) and linked to “Lipid modification” and “trophectoderm cell differentiation” GO terms, exhibited specific induction and stable expression in TELSCs across various passages (Figs. 1H, 1I, and S1L–O; Table S1). Gene set enrichment analysis (GSEA) further revealed that both TELCs and TELSCs exhibited significant enrichment of placental development-related pathways, underscoring their functional resemblance to *in vivo* TE lineages (Fig. S1P).

A recent study claimed to capture a novel type of TESCs, which resembled E4.5 polar TE lineages (Seong et al., 2022). We next compared the TELCs and TELSCs we captured in this study with TESCs, as well as the well-known TSCs cultured in both traditional serum-containing TS medium and optimized serum-free TX medium, at the transcriptomic level. We found that distinct from TELCs or TELSCs we captured in this study, TSCs exhibited obvious post-implantation E5.5–6.5 ExE characteristics, with high expression of ExE-specific genes, such as *Prl2c5*, *Serpinb9e*, *Rangrf*, and *Dnmt3b* (Fig. 1H and 1I). Interestingly, although we indeed detected 202 genes, including *Cited2*, *Pdgfa*, *Hand1*, *Bmp8b*, and *Gsto1*, were enriched in both TESCs and TELSCs cultured *in vitro*, as well as in E4.5 TE cells *in vivo*, TESCs were particularly enriched with 781 genes, such as *Rho5*, *Phlda2*, *Hmgn1*, and *Tcf7l2*, specifically expressed in E5.5–6.5 ExE tissues, which were not expressed in TELSCs. In contrast, there were 432 genes, including *Hspd1*, *Amotl2*, *Krt7*/*8*/*18*, and *Tspan8*, specifically enriched in E4.5 TE and TELSCs, which were not detected in TESCs (Fig. 1H and 1I). Thus, we consider that TESCs are dual-featured cells possessing both pre-implantation E4.5 TE and post-implantation E5.5–6.5 ExE characteristics. Comparably, TELSCs represent a novel type of TE-like stem cells with pure E4.5 TE features.

To provide a precise comparative analysis of these distinct TE-like cell populations in relation to *in vivo* embryonic development, we performed clustering analysis based on whole-transcriptome profiles. This analysis clearly demonstrated that TELCs clustered closely with E3.5 TE cells, while TELSCs exhibited transcriptional similarities to E4.5 TE cells, reflecting their resemblance to pre-implantation TE lineages (Fig. 1J). In contrast, both TESCs and TSCs showed global transcriptomic profiles more closely aligned with post-implantation ExE cells at E5.5–6.5 stages (Fig. 1J). The above result was consistent with and further supported by gene set variation analysis (GSVA) based on differentially expressed genes (DEGs) in TE3.5-ExE6.5 cells (Fig. 1K; Table S2). In summary, our findings indicate that distinct from previously reported TESCs or conventional TSCs, TELCs and TELSCs exhibit a resemblance to TE cells from E3.5 and E4.5 embryos during the pre-implantation stage, respectively, at the transcriptomic level.

### The unique epigenomic features of TELCs and TELSCs distinct from TSCs

**Figure 2.**
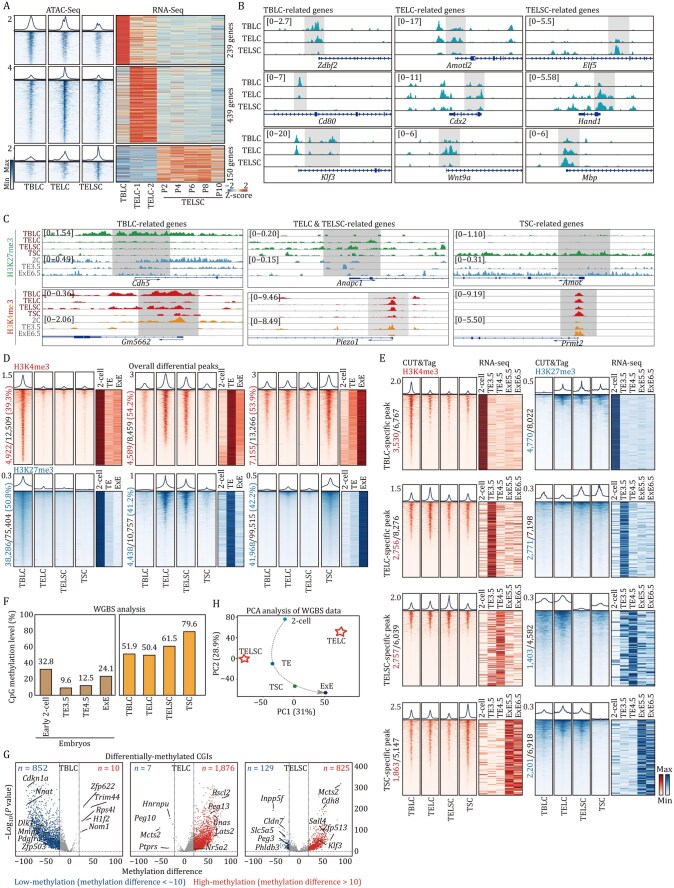
**The unique epigenomic features of TELSCs**. (A) ATAC-seq of TBLCs, TELCs, and TELSCs. The left heatmaps show the open or closed peaks around transcription start sites (TSSs) in TBLCs, TELCs, and TELSCs, and the relative expression of related genes based on RNA-seq on the right heatmap (RNA-seq data of TBLCs is from Shen et al. (2021)). (B) IGV browser view displaying ATAC-seq signals of specific genes in TBLCs, TELCs, and TELSCs. (C) IGV tracks showing H3K4me3 and H3K27me3 enrichment at representative marker genes across TBLCs, TELCs, TELSCs, and TSCs. (D) Left heatmap displaying the overall differential peaks across the genome between TBLCs, TELCs, TELSCs, and TSCs. Right heatmap showing the changes of differential peaks in mouse embryos (Andrews et al., 2023; Liu et al., 2016). (E) Heatmaps displaying the overall H3K4me3 and H3K27me3 differential peaks across the genome of between TBLCs, TELCs, TELSCs, and TSCs. Heatmaps on the right show the changes in the expression of these genes in mouse embryos data *in vivo* (Cheng et al., 2019; Sun et al., 2021; Wang et al., 2018a). (F) Histograms showing the global average methylation levels of TBLCs, TELCs, and TELSCs and mouse embryos based on WGBS data. The global average methylation levels of mouse embryos are from Sun et al. (2021) and Wang et al. (2018a). (G) WGBS analysis of TBLCs, TELCs, and TELSCs. Volcano plots displaying the differentially methylated CpG islands in TBLCs, TELCs, and TELSCs, respectively. For volcano plots, blue and red dots indicate low-methylation (methylation difference < −10) and high-methylation (methylation difference > 10) CpG islands of related genes with a *P*-value less than 0.05. WGBS data of TBLCs is from Shen et al. (2021). (H) PCA analysis of TELCs, TELSCs, TSCs, and mouse 2-cell, TE, and ExE based on WGBS data. WGBS data of TSCs is from Weigert et al. (2023), and WGBS data of mouse TE and ExE is from Wang et al. (2018a).

We next thought to characterize the epigenetic features of TELCs and TELSCs, and we performed transposase-accessible chromatin sequencing (ATAC-seq), cleavage under targets and tagmentation (CUT&Tag) for histone modifications, and whole-genome bisulfite sequencing (WGBS) to assess chromatin accessibility, histone modification patterns, and DNA methylation status, respectively. Notably, ATAC-seq analysis targeting transcription start sites (TSSs) revealed ATAC-seq signals in the promoter regions of 239 totipotent genes, including *Zdbf2*, *Cd80*, and *Klf3*, which were highly expressed in TBLCs but exhibited a pronounced decrease in TELCs and complete silencing in TELSCs, indicating a distinct open status in TBLCs that evidently transitioned to a closed status in TELSCs (Figs. 2A, 2B and S2A). Furthermore, in the promoters of 439 genes, such as *Amotl2*, *Cdx2*, and *Wnt9a*, which are selectively activated in TELCs, a TELC-specific chromatin open status was evident. Lastly, in the promoters of 150 genes, including *Elf5*, *Hand1*, and *Mbp*, particularly expressed in TELSCs, ATAC-seq signals gradually opened during the transition from TBLCs to TELSCs (Fig. 2A and 2B).

Next, a genome-wide comparison of histone modification, including H3K4me3 and H3K27me3, across gene bodies with *in vivo* 2C, TE, and ExE6.5 stages (Andrews et al., 2023; Liu et al., 2016) revealed that approximately half of the peaks in TBLCs, TELCs/TELSCs, and TSCs mirrored those in their corresponding *in vivo* counterparts (Fig. 2C and 2D). In total, we identified 3,530 and 4,770 unique H3K4me3 and H3K27me3 peaks, respectively, in TBLCs, which were absent in trophoblast cells and correlated with genes highly expressed in TBLCs. TELCs and TELSCs harbored 2,756 and 2,757 H3K4me3 peaks, along with 2,771 and 1,403 H3K27me3 peaks, respectively, corresponding to genes upregulated in these populations. In TSCs, 1,863 H3K4me3-enriched regions and 2,201 regions with H3K27me3 depletion were identified, associated with genes predominantly expressed in ExE5.5/6.5 cells *in vivo* (Fig. 2E). Representative totipotency markers such as *Gm5662* exhibited H3K4me3 enrichment specifically in TBLCs, while TELCs and TELSCs showed strong H3K4me3 signals at *Sirt1*, a gene essential for TSC differentiation and placental development (Arul Nambi Rajan et al., 2018). In contrast, *Amot*, a Hippo pathway member, was marked by H3K27me3 in TSCs but not in TELCs or TELSCs, suggesting a failure of TSCs to retain pre-implantation epigenetic features (Seong et al., 2022) (Fig. 2C).

Finally, WGBS analysis clearly showed that TELCs and TELSCs do not exhibit increased global DNA methylation levels compared to TBLCs. In contrast, TSCs exhibit significantly higher global DNA methylation levels than TBLCs, TELCs, or TELSCs (Fig. 2F). Additionally, the DNA methylation levels of many imprinted genes remain relatively stable without significant changes in TBLCs, TELCs, and TELSCs, which were much lower than those in TSCs (Fig. S2D). Subsequent analysis of methylation status on CpG islands (CGIs) showed that CGIs on the promoters of 852 genes lacking DNA methylation in TBLCs displayed clear methylation in TELCs and TELSCs, suggesting a cell fate specification process from totipotent stem cells toward the TE lineage (Fig. 2G). Additionally, we detected that TE-specific CGIs related to 1,876 genes (including *Peg13*, *Bscl2*, and *Gnas*) and 825 genes (containing *Cdh8*, *Sall4*, and *Zfp513*) were particularly methylated in TELCs and TELSCs, respectively. Additionally, we detected CGIs on the promoters of seven genes (including *Peg10*, *Mcts2*, and *Hnrnpu*) and 129 genes (containing *Peg3*, *Inpp5f*, and *Phldb3*) that were demethylated and transcriptionally induced, particularly in TELCs and TELSCs, respectively (Fig. 2G), illustrating TE-specific gene activation during TELC induction from TBLCs. PCA analysis using DNA methylation data revealed epigenetic similarities between TSCs and ExE6.5, while TELCs and TELSCs more closely resembled earlier stage TE (Fig. 2H).

In conclusion, the above comprehensive analysis elucidates the unique epigenetic features of TELCs and TELSCs that markedly differ from those of known TSCs.

### TELSCs specifically contribute to placental tissue with robust developmental potency to produce all trophoblast lineages

**Figure 3.**
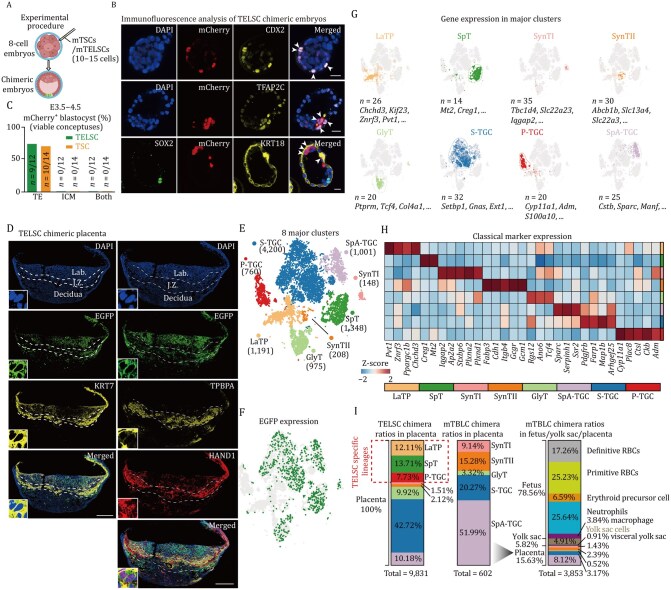
**TELSCs exhibit robust *in vivo* developmental potential and full trophoblast lineage contribution**. (A) Schematic of the experimental procedure to generate chimeric blastocysts. 10–15 EGFP-labeled TELSCs were injected into 8C-stage embryos. (B) Representative immunostaining of chimeric blastocysts injected with mCherry-labeled TELSCs. CDX2, TFAP2C, and KRT18: TE-speciﬁc markers; SOX2: ICM-specific marker; white arrows, TELSCs contribute to TE. Scale bar, 20 µm. (C) Bar graph showing the chimerism ratio between TELSCs and TSCs. (D) Immunofluorescence analysis of placenta sections from E12.5 chimeric placentae. The placenta was stained with the KRT7, TPBPA, HAND1, and also EGFP antibody to amplify the fluorescent signal. Lab., labyrinth; J.Z., junctional zone. The insets show enlarged images of the white boxes. Scale bar, 1 mm. (E) t-SNE plot showing the eight main trophoblastic clusters. (F) t-SNE plot showing EGFP expression in single EGFP^+^ cells from a chimeric placenta based on scRNA-seq. EGFP^+^ cells were sorted by flow cytometry. Green dots represent cells in which EGFP expression was detected in scRNA-seq. (G) t-SNE visualization showing marker gene expression in major clusters. (H) Heatmap showing the average expression of specific marker genes for each trophoblastic cluster. (I) Histogram indicating the proportion of cell types in TELSCs chimeric placentae and mTBLCs chimeric embryos.

To precisely assess the developmental potency of TELSCs, we first performed *in vivo* embryo chimerism assays by injecting mCherry- or enhanced green fluorescent protein (EGFP)-labeled TELSCs or TSCs into donor eight-cell embryos. TELSCs consistently contributed to the TE lineage from early to fully hatched blastocyst stages, similar to TSCs (Figs. 3A and S3A). Immunostaining analysis further confirmed that TELSCs efficiently and specifically integrated into the TE lineage, marked by CDX2 and KRT18, but not the embryonic epiblast lineage labeled by SOX2 (Figs. 3B, 3C, S3B and S3C). At the later developmental stage around E13.5, we detected that TELSCs can widely contribute to placental tissues, including both the junctional zone (JZ) and labyrinth (Lab) regions, but not yolk sac or fetal tissues (Fig. S3D), showing the developmental specificity of TELSCs toward trophoblast lineages. Further immunohistochemistry analysis clearly demonstrated TELSCs produced various trophoblast lineages marked by the general trophoblast marker KRT7, as well as lineage-specific markers HAND1 and TPBPA, indicative of their differentiation into TGCs and SpTs, respectively (Fig. 3D).

To further precisely elucidate the differentiation potential of TELSCs, we performed scRNA-seq on placental tissues from E13.5 chimeric mice. The results provide clear and direct evidence that TELSCs contribute to all eight trophoblast lineages reported to date, including Lab trophoblast progenitors (LaTPs), trophoblast glycogen cells (GlyTs), sinusoidal trophoblast giant cells (S-TGCs), spiral artery-associated trophoblast giant cells (SpA-TGCs), parietal trophoblast giant cells (P-TGCs), SpTs, and syncytiotrophoblast layer I (SynTIs) and layer II (SynTIIs) (Figs. 3E–H and S3E). Notably, TELSCs showed substantial contribution to SpA-TGCs, a specialized trophoblast subtype essential for forming the JZ and mediating maternal–fetal communication (Fig. 3E and 3G). While we further compared the cell lineage contribution of original TBLCs with that of TELSCs in the same chimeric assay (Peng et al., 2025), we found that compared to TBLCs, which can widely contribute to various embryonic and extraembryonic cells, TELSCs specifically generate trophoblast lineages, but not any other embryonic cell types, therefore proving the very specific developmental potency of TELSCs, which eventually led to the widespread contribution of TELSCs to all trophoblast lineages (Fig. 3I).

Nevertheless, for the very first time, our study directly and comprehensively demonstrates that TELSCs are able to produce all the trophoblast lineages *in vivo* at the single-cell resolution, since previous studies on TSCs, mainly based on immunostaining analysis, could not provide such definitive evidence, which therefore provides new criteria to precisely evaluate and compare the cell lineage contributions of various kinds of trophoblast-like stem cells.

### TELSCs exhibit superior trophoblast differentiation capacity compared to TSCs in the mouse teratoma assay

**Figure 4.**
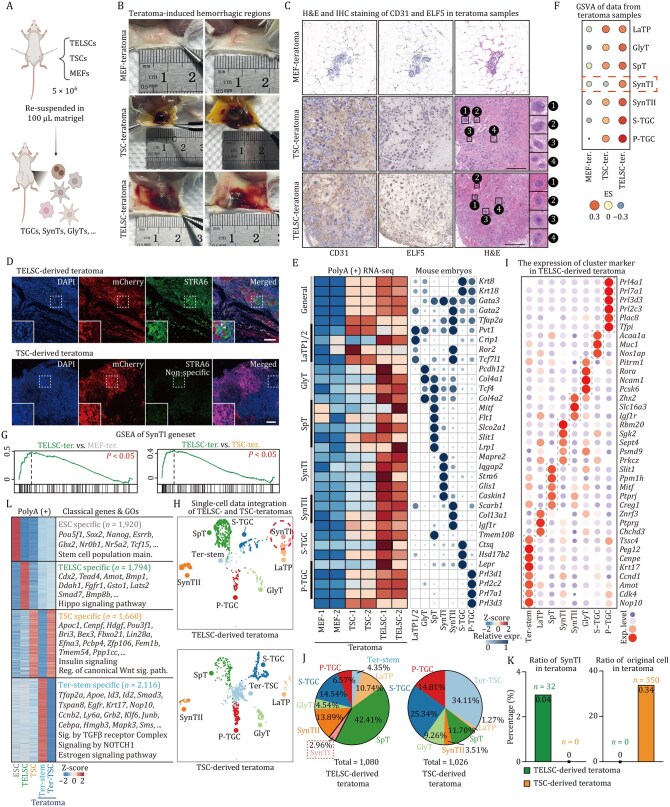
**TELSCs exhibit enhanced *in vivo* trophoblast differentiation potential compared to TSCs in teratoma assays**. (A) Schematic view for the teratoma-forming assay. MEFs, TSCs, and TELSCs were re-suspended and subcutaneously injected into 6- to 8-week-old female NOG mice. (B) TSCs, TELSCs, or control MEFs were injected subcutaneously into both flanks of NOG mice. Lesions were analyzed 10 days after injection. (C) Teratoma was dissected and fixed by H&E staining and IHC (Immunohistochemical) staining against the trophoblast marker ELF5 and endothelial marker CD31. The right image shows the morphological characteristics of TGCs with the enlargement of nuclei. (D) IF (Immunofluorescence) staining of teratoma tissues derived from TELSCs and TSCs. STRA6: SynTI-speciﬁc marker. Scale bars, 200 µm. (E) Heatmap (left) and bubble chart (right) showing relative expression of the indicated genes in teratoma derived from MEFs, TSCs, and TELSCs. Trophoblastic lineages data are from Jiang et al. (2023). (F) GSVA analysis of teratoma derived from MEFs, TSCs, and TELSCs. (G) GSEA analysis of teratoma from MEFs, TSCs, and TELSCs based on SynTI geneset. (H) Uniform Manifold Approximation and Projection (UMAP) visualization showing cells from TELSCs and TSCs teratomas colored by cell types. (I) Dot plots indicating the expression of cluster-specific genes in teratoma from TELSCs. (J) Pie chart indicating the proportion of cell types from TELSCs and TSCs teratomas. (K) Histogram indicating the ratio of SynTI from TELSCs and TSCs teratomas and the ratio of the original cells in teratomas derived from TELSCs and TSCs. (L) Heatmap showing DEGs among TELSCs, TSCs, TELSC-derived Ter-stem cells isolated from teratomas, and residual TSC-like cells (Ter-TSC) present in TSC-derived teratomas. Representative marker genes and enriched Gene Ontology (GO) terms are highlighted.

Beyond the above chimerism assay, we also conducted a teratoma analysis by injecting the same amount of TELSCs, TSCs, and mouse embryonic fibroblasts (MEFs, as a negative control) into immunodeficient nude mice, respectively (Fig. 4A). We observed that both TSCs and TELSCs can efficiently form teratoma tissues, which can cause hemorrhagic lesions characterized by large blood-filled lacunae, but MEFs cannot (Figs. 4B and S4A). Further histological analysis of these lesions revealed a typical trophoblastic hemorrhagic structure with ELF5-positive TGCs that were differentiated from TELSCs or TSCs (Fig. 4C), clearly showing the invasive properties of TGCs. An immunohistofluorescence assay further confirmed the presence of trophoblasts expressing KRT7 and PEG10, including SpTs and TGCs marked by TPBPA and PRL, respectively, in both TELSC- and TSC-derived teratomas (Fig. S4B), demonstrating the trophoblast developmental potency of both TELSCs and TSCs.

To precisely assess the cell fate commitments of TELSCs and TSCs, we performed qPCR, bulk RNA-seq, and scRNA-seq analyses. These analyses clearly showed that, compared with the control sample, a large number of trophoblast-specific, but not embryonic lineage-specific, marker genes, were activated in both TSC- and TELSC-derived teratoma tissues, showing the TE developmental specificity of TELSCs (Figs. 4E, 4H, S4C and S4D). Interestingly, bulk RNA-seq and qPCR analysis showed that the expression of a large group of mature trophoblast-specific genes, including *Crip1* (a marker for LaTP), *Serpinh1* (GlyT), *Glis1* (SynTI), *Col13a1* (SynTII), *Ghrh* (S-TGCs), *Flt1* (SpT), and *Prl2c2* (P-TGCs), was much higher in TELSC-derived teratomas than in the TSC-derived ones (Figs. 4E and S4E; Table S3). Further GSEA and GSVA analyses consistently highlighted the superior trophoblast differentiation capacity of TELSCs, especially their unique ability to generate SynTI cells, a lineage not observed in TSC-derived teratomas (Fig. 4F and 4G; Table S4), as further confirmed by immunofluorescence staining of teratomas (Fig. 4D).

Precisely, scRNA-seq demonstrated that both TELSCs and TSCs gave rise to various trophoblast lineages, except for SpA-TGCs—known to require maternal signaling cues within the placenta (Figs. 4H, 4I, S4F and S4G). While compared to TELSCs that can produce all other trophoblast lineages, TSCs failed to generate SynTI cells and rarely produced LaTP cells, which was quite consistent with RNA-seq and qPCR analysis (Figs. 4H, 4J, 4K and S4C). Interestingly, we observed that a large group of undifferentiated TSCs, marked by *Rangrf*, *Nap1l1*, *Fbo21*, and *Efna3*, remained in TSC-derived teratoma tissues. In comparison, undifferentiated TELSCs were barely detectable in TELSC-derived ones, indicating that TELSCs have much higher differentiation potential than traditional TSCs (Fig. 4H and 4K). This observation was quite consistent with qPCR and RNA-seq analysis, which showed significantly higher expression levels of stem cell marker genes in TSC-derived teratomas than in TELSC-derived ones (Fig. S4C and S4H). In contrast, within TELSC-derived teratomas, we identified a unique group of ExE-like cells, marked by *Elf5*, *Hspd1*, *Krt17*, and *Amot*, accounting for approximately 4% of the total cell population, which could not be detected in TSC-derived tissues, and we named this population “Ter-stem” (Figs. 4H, 4I, 4J and S4F). Interestingly, we detected 2,116 genes related to the TGF-beta and Notch signaling pathway, including *Tfap2a*, *Apoe*, *Smad3*, and *Krt17*, that were specifically enriched in these Ter-stem cells, but not in TSCs. Comparatively, teratoma-retained undifferentiated TSCs were highly enriched with 1,660 genes related to Wnt signaling pathways, which were highly expressed in original TSCs, yet could not be detected in TELSC-derived Ter-stem cells in teratoma tissue (Fig. 4L). Thus, TELSC-derived Ter-stem cells in teratoma tissue were distinct from known TSCs.

Collectively, in the teratoma assay, TELSCs displayed higher trophoblast developmental potency than TSCs and produced almost all mature trophoblast lineages and a unique population of ExE-like Ter-stem cells distinct from TSCs.

### TELSCs can efficiently generate trophoblast organoids with mature trophoblast lineages and self-renewal ability

**Figure 5.**
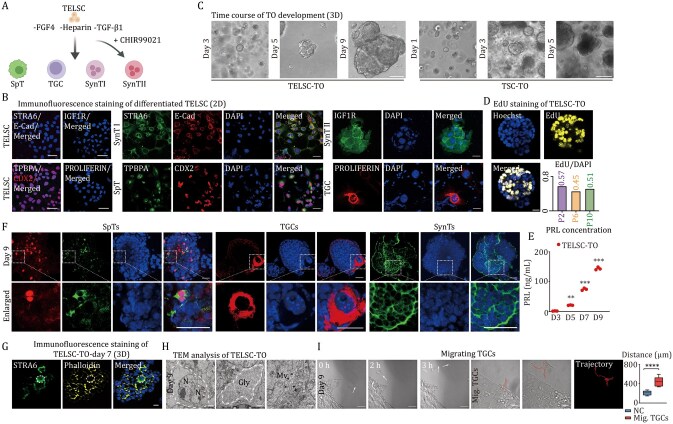
**TELSCs, but not TSCs, can efficiently generate trophoblast organoids with mature trophoblast lineages and self-renewal ability**. (A) Schematic of the procedures used to induce downstream differentiation of TELSCs in 2D culture conditions. (B) Immunofluorescence staining of marker genes of downstream trophoblast cells (STRA6, IGF1R, TPBPA, and PROLIFERIN) in differentiated TELSCs. Scale bars, 50 µm. (C) Morphology of trophoblast organoids derived from TELSCs and TSCs at different time points. Scale bars, 100 µm. (D) EdU staining of long-term cultured TELSC-TO. Scale bars, 25 µm. The ratio of EdU/DAPI is shown in the lower right. (E) ELISA analysis of prolactin (PRL) in the medium secreted by TELSC-TO from Day 3 to Day 9 (*n *= 3). (F) Immunofluorescence staining of trophoblast organoids. TPBPA for SpT, PROLIFERIN for TGC, and STRA6 for SynTI. Scale bars, 50 µm. (G) Immunofluorescence staining of trophoblast organoids showing the STRA6^+^ multinucleate cell. Scale bars, 20 µm. (H) Transmission electron microscope (TEM) view of TELSC-TO. Multinucleate cells, glycogen, and microvilli were shown. N, nucleus; Gly, glycogen; Mv, microvillus. (I) The migration path of TGCs from TELSC-TO and quantification of the migration distance of TGCs. Scale bars, 25 µm. Mean ± SD; *n *= 10; *P < 0.05, **P < 0.01, ***P < 0.001, ****P < 0.0001, unpaired Student’s *t*-test.

Next, we tried to assess the differentiation capacity of TELSCs *in vitro* and the related application in TO formation. We first constructed spontaneous differentiation by plating TELSCs in TX medium lacking FGF4, Heparin, and TGFβ1 for approximately 9 days, and we observed discernible morphological transformations toward trophoblast lineages (Figs. 5A and S5A). qRT-PCR analysis revealed trophoblast lineage-specific genes, including *Syna* and *Plxnd1* for SynTI, *Synb* and *Gcm1* for SynTII, *Ascl2* and *Tpbpa* for SpT, and *Prl2c2* and *Ctsq* for TGC, underwent gradual and pronounced activation during the directed differentiation (Fig. S5B). Further immunostaining analysis confirmed the appearance of corresponding lineages, such as SynTI (labeled by STRA6 and E-CADHERIN), IGF1R for SynTII, SpT (labeled by TPBPA and CDX2), and TGC (labeled by PROLIFERIN), after 9 days of induction (Fig. 5B). Hence, it can be concluded that TELSCs possess the capability to generate diverse trophoblast lineages under the withdrawal of FGF4, Heparin, and TGFβ1 in two-dimensional (2D) culture conditions.

The placenta, composed of diverse trophoblast lineages, plays a crucial role in fetal-maternal communication, yet the *in vitro* model mimicking the entire placenta development was still lacking. We then tested the possibility of deriving TOs from TELSCs by plating TELSCs, along with TSCs as the control, into modified human placental organoid culture medium, containing murine FGFβ, HGF, and EGF (Haider et al., 2018; Karvas et al., 2022; Turco et al., 2018) for Matrigel-based 3D culture. After 3 days, we found the formation of organoid-like structures from TELSCs, which exhibited sustained and progressive growth over a period of at least 9 days (Fig. 5C). In contrast, TSC-derived structures displayed impaired development with significant apoptosis after Day 3 (Fig. 5C). By Day 9, TELSC-derived TOs developed into dense, solid masses. TUBULIN immunostaining, highlighting the cytoskeleton, confirms their well-organized, maturing structure (Fig. S5C). Notably, these TELSC-derived TOs can be maintained through long-term passages (Fig. S5D). While the EdU incorporation assay revealed the presence of proliferating cells across different passages, supporting the self-renewal capacity of TELSC-TOs (Figs. 5D and S5E).qRT-PCR analysis revealed a rapid decrease of TELSC-specific genes (*Cdx2*, *Elf5*, and *Eomes*) after 3 days, indicating the initiation of differentiation. Correspondingly, trophoblast lineage markers, including *Syna* for SynTI, *Synb* and *Gcm1* for SynTII, *Ascl2* and *Tpbpa* for SpT, *Prl2c2* and *Ctsq* for TGC, exhibited dramatic induction after 7 days, signifying trophoblast specification (Fig. S5F). While immunostaining analysis clearly showed that these TELSC-derived TOs, up to 10 passages, encompass various trophoblast lineages, such as SpT (TPBPA), TGC (PROLIFERIN), and SynTI (STRA6) (Figs. 5F, 5G and S5G–K). Using transmission electron microscopy (TEM), we detected the trophoblast cells with multiple nuclei sharing a continuous cytoplasm without intervening membranes, and the well-developed microvilli (Mv), representing SynT cells in TELSC-derived TOs. In addition, we observed glycogen granules (Gly) probably in the TGCs (Fig. 5H). Notably, TGCs with large nuclei gathered together surrounding the TOs and seemed able to migrate under living cell imaging (Fig. 5I). Over 30% of these organoids also exhibited trophoblast outgrowth, mimicking placental invasion *in vivo* (Fig. S5L and S5M). It has been known that TGCs can produce prolactin (PRL), a hormone essential for pregnancy and the production of breast milk, and we then quantified PRL production in the placental organoids containing TGCs. We detected that TELSC-derived organoids gradually released increasing levels of PRL, reaching up to 150 ng/mL on the ninth day (Fig. 5E). Thus, TELSCs can be widely used for trophoblast differentiation and TO generation formation.

### Newly identified ExE-like progenitors enable coupled self-renewal and differentiation abilities of TELSC-derived organoids

**Figure 6.**
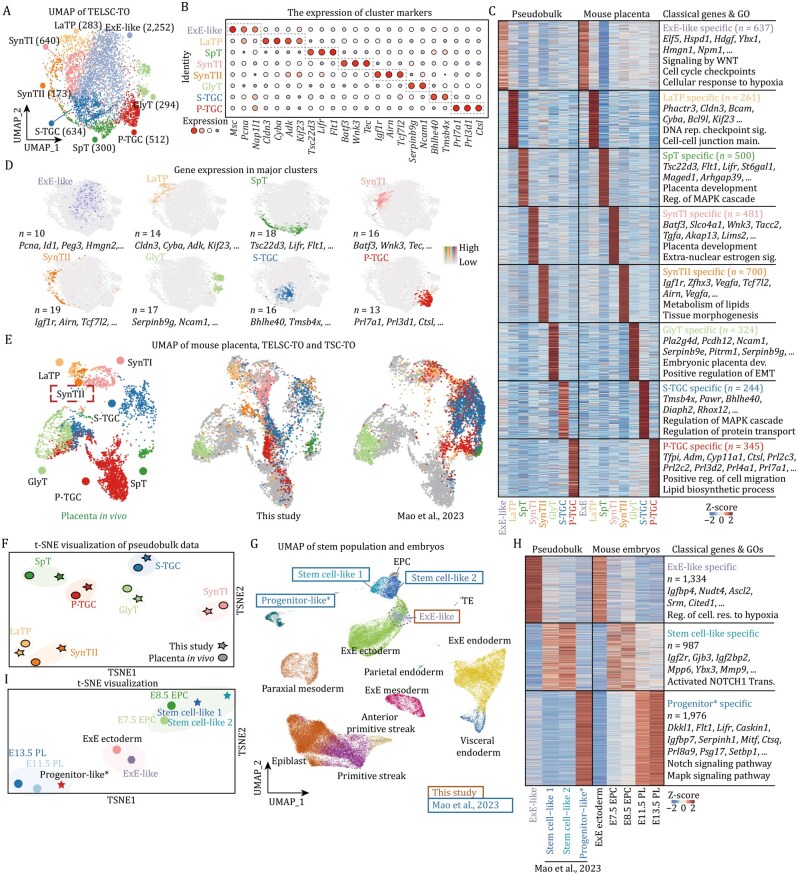
**Newly identified ExE-like progenitors enable coupled self-renewal and differentiation abilities of TELSC-derived organoids**. (A) Cellular composition of mouse TELSC-derived TO revealed by scRNA-seq. UMAP plot showing the eight main clusters. (B) Dot plots indicating the expression of cluster-specific genes in TELSC-TO. (C) The heatmap displays the top differentially expressed genes across each identified cluster. Cluster-specific marker genes and representative Gene Ontology (GO) terms are listed on the right. (D) UMAP visualizations showing the expression of the marker genes in major clusters. (E) UMAP visualizations of the single-cell transcriptome of cells from TELSC-TO and the reported mouse trophoblast organoid integrated with the mouse placenta. (F) t-SNE analysis of each cell type from TELSC-TO and the mouse placenta. (G) Integration of stem cell population from TELSC-TO (ExE-like), TO reported by Mao et al. (2023) and mouse embryo (Posfai et al., 2021). (H) Heatmap on the left demonstrating the DEGs between ExE-like from TELSC-TO and the stem cell population from Mao et al. (2023). Heatmap on the right demonstrating the expression of each cluster in mouse embryos. (I) t-SNE analysis of stem cell population from TELSC-TO and the reported mouse trophoblast organoid.

To uncover the comprehensive cell lineages within TELSC-derived TOs, we performed scRNA-seq analysis on Day-9 TOs derived from TELSCs and obtained approximately 5,088 single cells of high quality (Fig. 6A). This analysis clearly showed that TELSC-derived organoids contained almost all reported differentiated trophoblast subpopulations (Fig. 6B and 6C), including 640 SynTI cells (marked by *Batf3*, *Wnk3*, and *Tec*), 173 SynTII cells (marked by *Ank*, *Arhgap44*, and *Airn*), 283 LaTPs (marked by *Cldn3*, *Cyba*, and *Adk*), 300 SpTs (marked by *Maged1*, *Dapk2*, and *Tmem40*), 294 GlyTs (marked by *serpinb9e*, *Pitrm1*, and *Car2*), 634 S-TGCs (marked by *Hand1*, *Tmsb4x*, and *Bhlhe40*), and 300 P-TGCs (marked by *Prl3d1*, *Prl7a1*, and *Ctsl*) (Figs. 6A–D and S6A; Table S5).

In a recent study, Mao et al. (2023) claimed to generate TOs from TSCs; however, in which two different culturing media were required to maintain the proliferation of placenta organoids and to induce trophoblast differentiation, respectively. Thus, these TSC-derived TOs are defective and distinct from TELSC-derived ones with coupled self-renewal and differentiation abilities that we developed in this study. To precisely compare the cellular compositions and understand the differences of these two TO types, we next performed integration analysis using scRNA-seq data from our TELSC-derived or published TSC-derived organoids, as well as the *in vivo* mouse placenta (Jiang et al., 2023). This analysis clearly showed that TELSC-derived organoids contained almost all reported differentiated trophoblast subpopulations, which were highly comparable to the corresponding lineages in placenta tissue *in vivo*. Comparably, the TSC-derived TOs maintained in the trophoblast differentiation medium lacked the SynTII lineage (Figs. 6E and S6B). Besides, the transcriptome-based t-SNE analysis clearly showed that various trophoblast cells in TELSC-derived TOs can be aligned well with those corresponding cell lineages in the placenta tissues *in vivo* (Fig. 6F).

Interestingly, in the TELSC-derived organoids, we identified a unique, large population of 2,252 trophoblast progenitor cells, taking around 50% of all single cells obtained, which were enriched with typical trophoblast progenitor marker genes, such as *Fabp3*, *Igfbp4*, *Srm*, and *Pdgfa*, which were highly enriched in E5.5–6.5 ExE cells (Fig. 6A and 6D). We therefore named these cells ExE-like progenitor cells. Further transcriptome-based clustering analysis clearly showed these ExE-like progenitor cells were indeed comparable to ExE ectoderm at E5.5–6.5, but not other stages (Fig. 6G). Interestingly, stem cell-like and trophoblast progenitor-like populations were also identified in TSC-derived TOs cultured in the maintenance medium, as recently reported. We therefore compared these TSC-derived stem cell-like populations, which were specially enriched with 987 genes, such as *Gjb3*, *Igf2bp2*, *Mpp6*, *Ybx3*, and *Mmp9*, which were particularly expressed in E7.5-8.5 EPC cells. Whereas, TSC-derived trophoblast progenitors particularly expressed 1,987 genes, including *Flt1*, *Lifr*, *Caskin1*, *Serpinh1*, *Prl8a9*, and *Setbp1*, which were enriched in mature trophoblast lineages at around E11.5–13.5 stage. Comparably, there were 1,334 genes specifically enriched in TELSC-derived ExE-like progenitor cells, which were highly expressed in E5.5–6.5 ExE ectoderm cells *in vivo* (Figs. 6H, S6C and S6D). Further single-cell-based integration and t-SNE analysis clearly showed that TSC-derived stem cell-like and progenitor-like populations resembled E7.5–8.5 EPC cells and E11.5–13.5 trophoblast lineages, respectively. While TELSC-derived ExE-like progenitors were close to E5.5–6.5 ExE ectoderm cells *in vivo* (Fig. 6I).

Finally, we noticed that ExE-like cells displayed elevated expression of proliferation-associated genes, suggesting a higher proliferative capacity and enhanced stemness compared to the stem cell-like and progenitor-like populations in TSC-derived TOs (Fig. S6E and S6F). Summarily, TELSC-derived organoids with comprehensive mature trophoblast lineages and unique E5.5–6.5 ExE-like progenitors are clearly distinct from TSC-derived ones reported recently. These ExE-like cells with high proliferation capacity allow the coupled self-renewal and differentiation abilities of TELSC-derived organoids in a uniform medium for long-term passages.

### The rosette structure, relying on ITGB1, is required for ExE-like progenitor induction and TELSC-derived TO formation

**Figure 7.**
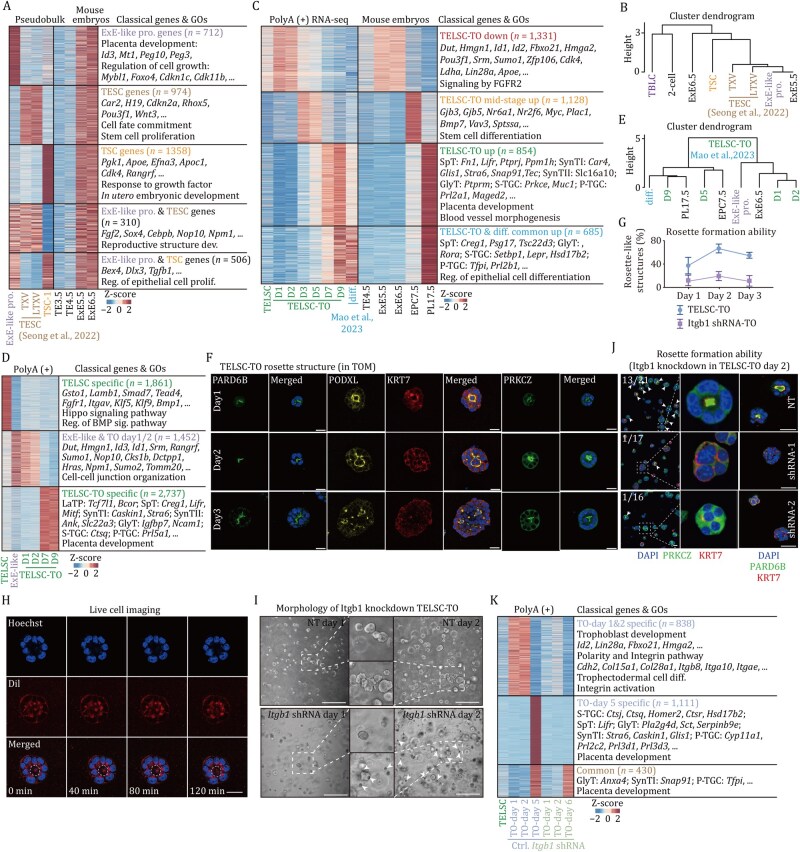
**The rosette structure, relying on ITGB1, is required for ExE-like progenitor induction and TELSC-derived TO formation**. (A) Heatmap on the left demonstrating the DEGs between stem cell population in TELSC-TO (ExE-like), TESCs, and TSCs. Heatmap on the right demonstrating the expression of each cluster in mouse embryos. (B) Hierarchical clustering analysis of ExE-like, TESCs, TSCs, TBLCs, and mouse embryos data. (C) Heatmap indicating the differentially expressed genes of TELSC-TO, TSC-TO, and TO reported by Mao et al. (2023) and mouse embryo (Cheng et al., 2019; Koppes et al., 2019; Sun et al., 2021; Tuteja et al., 2016). The classical genes and enrichment of GO terms of these genes are shown on the right. (D) The heatmap illustrates distinct transcriptional signatures among TELSCs, ExE-like cells identified in single-cell RNA-seq of TELSC-TOs, and bulk RNA-seq profiles of TELSC-TOs. Cluster-specific marker genes and representative GO terms are listed on the right. (E) Hierarchical clustering was performed based on transcriptomic profiles from the ExE-like population identified in TELSC-derived TOs (both single-cell and bulk RNA-seq), mTOs reported by Mao et al. (2023), and corresponding *in vivo* mouse embryos. (F) Immunofluorescence staining of PARD6B, PODXL, KRT7, and PRKCZ in TELSC-TO from Day 1 to Day 3. Scale bars, 25 µm. (G) Line graph showing the percentage of TELSC-derived organoids that formed rosette-like structures at Days 1, 2, and 3. (H) Live-cell imaging of rosette-like structures in TELSC-TO. Scale bars, 25 µm. (I) Morphology of TELSC-TO after transfected with NT (non-targeting) and Itgb1 shRNA. (J) NT and *Itgb1* shRNA-1 to -2 were transfected into TELSCs. The location of PRKCZ and PARD6B in TELSC-TO was measured by immunofluorescence staining. Scale bars, 25 µm. Quantification of the number of TELSC-TO with rosette-like structures transfected with NT and *Itgb1* shRNA-1 to -2 was labeled. (K) Heatmap indicating the differentially expressed genes of TELSCs, TELSC-TO, and TELSC-TO transfected with *Itgb1* shRNA. The representative genes and enrichment of GO terms of these genes are shown.

Since these TELSC-derived ExE progenitor cells, TSCs, and recently reported TESCs all displayed ExE ectoderm features (Fig. 6G and 6H), we then performed a transcriptome comparison on these cells. In brief, there were 712 genes that were specifically related to placenta development and cell growth, including *Peg10*/*3*, *Id3*, *Foxo4*, *Cdkn1c*/*11b*, and *Mybl1*, highly expressed in the ExE-like cells and E5.5–6.5 ExE ectoderm cells, but not in reported TSCs or TESCs (Fig. 7A). Additionally, we detected 974 genes related to stem cell proliferation (exemplified by *Car2*, *H19*, *Pou3f1*, and *Wnt3*) and 1,356 genes associated with *in utero* embryonic development (such as *Pgk1*, *Apoe*, and *Apoc1*), all of which were specifically expressed in E5.6–6.5 ExE ectoderm cells and were specifically enriched in TESCs and TSCs, respectively. There were also 310 ExE-specific genes related to reproductive structure development, including *Fgf2*, *Sox4*, *Cebpb*, and *Nop10*, which were particularly highly expressed in both ExE-like progenitors and TESCs, but not TSCs (Figs. 7A and S7A). Thus, ExE-like cells exhibited novel E5.5–6.5 ExE features distinct from conventional TSCs or newly reported TESCs (Figs. 7B and S7B).

To further precisely understand the induction of ExE-like progenitors during TELSC-derived organoid formation, we performed RNA-seq on TELSC-derived organoids at various time points, including Days 1, 2, 3, 5, 7, and 9. More than 800 genes were specifically activated after 7–9 days in TELSC-based organoid formation, and these genes ­represented various trophoblast lineage-specific genes, including *Fn1* and *Mitf* for SpT, *Stra6* and *Car4* for SynTI, *Atxn1* and *Col13a1* for SynTII, *Col4a1*, and *Ptprm* for GlyT, *Arhgef25* and *Prkce* for S-TGC (Fig. 7C; Table S6), indicating eventual commitment to distinct mature trophoblast lineages. In addition, approximately 936 genes were specifically activated between Days 3 and 5, including *Gjb3* and *Mpp6*, which are primarily expressed during the embryonic EPC stage *in vivo*. Interestingly, a cluster of 645 genes was dynamically induced after 1–2 days but decreased rapidly after 3 days and was observed specifically during TELSC differentiation. Notably, these genes were associated with trophoblast progenitors, including *Dut*, *Id3*, *Hmgn1*, and *Id1*, which were also highly expressed in E5.5–6.5 ExE ectoderm, and ExE-like progenitors identified in TELSC-derived TOs (Fig. 7D). Further clustering analysis and GSEA indicated that these Day 1/2 cells closely resembled ExE-like progenitors in TELSC-derived TOs; Day 5 cells displayed transcriptomic features akin to those of the epc stage; whereas, in comparison, cells from Day 7–9 organoids showed transcriptomic profiles similar to those of mature placental tissues at E9.5 and E17.5 (Figs. 7E and S7C). Thus, ExE-like progenitors, representing an intermediate phase for trophoblast progenitor expansion, were induced at Days 1–2 during TELSC differentiation.

During *in vivo* mouse embryo development at around E5.5–6.5, both embryonic epiblast and extraembryonic ExE cells form a rosette-like structure with apical domains, showing an unpolarized-to-polarized transition, which is essential for lumenogenesis of developing embryos and the related cell differentiation process (Bedzhov and ­Zernicka-Goetz, 2014; Christodoulou et al., 2019; Lyu et al., 2024). Interestingly, immunostaining analysis using antibodies against PARD6B and PODXL, well-known apical domain markers, clearly showed that typical rosette-like structures with the expression of PARD6B and PODXL at the lumen center were transiently and dynamically induced on Days 1 and 2, yet disappeared after Day 3, during TELSC-derived organoid formation, regardless of whether our own culture system or that described by Mao et al. (2023) was used (Figs. 7F, 7G and S7D). Further live cell imaging using Dil staining at 0, 40, 80, and 120 min showed the rosette structure forms and maintains stability for a long time, indicating a stable cellular arrangement event (Fig. 7H). In contrast, TSC-derived organoids did not efficiently generate rosette structures like TELSCs under both culture conditions (Fig. S7E and S7F), showing the functional deficiency of TSCs on embryo-like structures compared to TELSCs. Since the induction of the above rosette-like structures and ExE-like progenitors occurred at the same time, we proposed that the rosette-like structure formation was essential for ExE-like progenitor cell fate determination.

To test the above hypothesis, using shRNAs, we knocked down the expression of *Itgb1*, a key integrin β1 signaling gene required for apical domain formation, in TELSCs and subsequently assessed their capacity to form rosette structures. qPCR was performed, and confirmed the efficient knockdown of *Itgb1*, which interestingly also significantly reduced mRNA levels of polarity markers, including *Pard6b*, *Podxl*, and *Prkcz*, in the cells after 2 days of induction for TO formation (Fig. S7G). Immunofluorescence staining further revealed a significant reduction in the proportion of rosette structures (Fig. 7I), eventually leading to peripheral cell death beginning after 2 days (Fig. 7J). RNA-seq was then performed on organoid samples from Day 1 to Day 6 post-shRNA treatment. As expected, we found ITGB1 knockdown obviously inhibited the dynamic activation of 838 genes, involved in extracellular matrix organization (e.g., *Col15a1*, *Col28a1*), cell adhesion (e.g., *Itgb8*, *Itga10*), and ExE-stage identity (e.g., *Id2*, *Lin28a*, and *Fbxo21*), which were highly enriched in ExE-like progenitors in TELSC-derived organoids and ExE cells *in vivo*, therefore inhibiting the induction of ExE-like cells at Days 1 and 2 (Fig. 7K). Consistently, GSEA analysis confirmed significant downregulation of pathways related to integrin signaling and cell polarity following ITGB1 knockdown (Fig. S7H). At later stages, these knockdown organoids failed to fully develop. RNA-seq analysis of Day 6 samples revealed reduced expression of multiple lineage-specific markers compared to the WT group, including *Ctsj* (S-TGC), *Lifr* (SpT), and *Pla2g4d* (GlyT), indicating that early disruption of rosette morphogenesis substantially impairs subsequent lineage specification (Fig. 7K).

Altogether, using the TELSC-derived organoid model, we demonstrate that the formation of rosette-like structures, relying on the key integrin β1 signaling factor *Itgb1*, is indispensable for ExE-like progenitor induction and further TO generation from TELSCs, which could explain the deficiency of TSCs in TO formation. Nevertheless, capturing TELSCs enables us to faithfully recapitulate key morphogenetic events of the entire trophoblast development *in vitro*, showing the widespread applications in basic studies and translational medicine.

## Discussion

In our recent study, we have successfully established the capture and long-term maintenance of human and mouse totipotent stem cells, TBLCs, comparable to two- and four-cell stage blastomeres, through spliceosomal repression (Li et al., 2024; Shen et al., 2021). Our further investigation into the differentiation potential of these cells toward various functional cells, particularly the extraembryonic lineages containing trophoblasts, highlights the unique developmental potency of TBLCs. Here, based on the Hippo-YAP/Notch-to-TGFβ1 signaling switch, we developed a “two-step” differentiation system to robustly and efficiently induce TBLCs or eight-cell blastomeres to produce a new kind of TELSCs, reassembling the E4.5 TE cells, which are distinct from the well-known TSCs or newly reported TESCs (Seong et al., 2022) and TSCs (Kubaczka et al., 2014). Importantly, TELSCs exhibit specific TE properties in both teratoma differentiation and chimera formation assays. We demonstrated that TELSCs can broadly produce all major placental trophoblast lineages, including LaTPs, GlyTs, S-TGCs, SpA-TGCs, P-TGCs, SpTs, SynTIs, and SynTIIs, at the single-cell level for the very first time. Therefore, our study provides a very useful platform and model to study the earliest events of trophoblast development from totipotent cells.

In our 3D culture system, TELSCs resembling TE4.5 transit through an E5.6–6.5 ExE-like phase before differentiating into mature TOs with diverse lineages. Interestingly, transcriptomic comparison showed that these ExE-like progenitors were clearly different from reported TSCs or TESCs, although both have partial features of E5.5–6.5 ExE ectoderm *in vivo*. Notably, the production of these ExE-like progenitors highly relies on the morphological change with the formation of rosette-like structures, which were governed by the key integrin β1 signaling gene, *Itgb1*. These progenitors with rapid cell proliferation capacity can be persistently retained in TELSC-derived organoids, which therefore enabled the formation of TOs with coupled self-renewal and differentiation abilities for long-term passages. Comparably, TSCs cannot undergo similar rosette structure transformation, and therefore cannot generate organoids in the same culturing condition, which might reflect the deficiency of conventional TSCs with post-implantation characters compared to TELSCs we captured in this study. In contrast, two types of culturing medium were newly developed and required to maintain the proliferation and differentiation of TSC-derived TOs that failed to produce mature SynTII cells, respectively.

Our newly established mouse TOs from TBLCs offer a comprehensive model for studying mouse placental development, facilitating high-throughput genetic screening and in-depth investigation into pre-, peri-, and post-implantation processes. By establishing the complete developmental path from totipotent cells to trophoblast cells, we are now able to monitor trophoblast developmental abnormalities starting from the totipotent stage. Furthermore, given that placenta-associated disorders are complex conditions that may involve dysregulation of maternal immune cells as well as defects in fetal-derived trophoblast development, the TOs we generated allow us to exclude variables related to maternal cells and focus specifically on trophoblast-specific mechanisms. Moreover, future efforts will include immunizing or vascularizing the organoids to further investigate placental disorders caused by maternal tissue abnormalities. Finally, gene editing technologies can be employed to generate mutant organoids carrying specific genetic alterations, thereby faithfully modeling genetic mutation-related placental diseases. This is pivotal for exploring the pathophysiological mechanisms underlying implantation failures and pregnancy disorders, such as miscarriage and preeclampsia, which are frequently attributed to defective placentation. This *in vitro* system provides critical insights into these reproductive disorders, advancing our comprehension of trophoblast differentiation from totipotent stem cells and providing a powerful platform for placental development research.

## Limitations of the study

While mouse TELSCs can be efficiently derived from TBLCs to form placental TOs, the application of this system, particularly for modeling pregnancy-related disorders in basic research and reproductive medicine, should be widely explored. The co-culture system containing the TELSC-derived TO, maternal decidual cells, as well as the immune and endothelial cells, needs to be further developed, which will be very important to fully understand the fetal-maternal communications during the establishment and maintenance of pregnancy. Due to undetermined sex of both donor and host embryos, the impact of sex-specific factors on trophoblast functionality and chimeric efficiency remains unassessed. While transcriptional profiles closely mimic *in vivo* trophoblasts, future studies will focus on functional assays, such as barrier assays and nutrient-transport measurements, to validate the physiological relevance of TELSC-TOs. Finally, whether the similar culture conditions can be applied to capture human TELSC and TELSC-derived organoids could be a very interesting research topic.

## Supplementary Material

pwaf098_Supplementary_Data

## Data Availability

The raw sequence data reported in this paper have been deposited in the Genome Sequence Archive (Genomics, Proteomics & Bioinformatics 2025) in National Genomics Data Center (Nucleic Acids Res 2025), China National Center for Bioinformation / Beijing Institute of Genomics, Chinese Academy of Sciences (GSA: CRA032439). [1] The GSA Family in 2025: A Broadened Sharing Platform for Multi-Omics and Multimodal Data. *Genomics, Proteomics & Bioinformatics* 2025 Sep 22;23(4):qzaf072. doi.org/10.1093/gpbjnl/qzaf072 [PMID=40857552] [2] Database Resources of the National Genomics Data Center, China National Center for Bioinformation in 2025. *Nucleic Acids Res* 2025 Jan 6;53(D1):D30-D44. doi.org/10.1093/nar/gkae978 [PMID=39530327]

## Abbreviations

ATAC-seq, transposase-accessible chromatin sequencing; CGIs, CpG islands; CUT&Tag, cleavage under targets and tagmentation; DEGs, differentially expressed genes; EPC, ectoplacental cone; ESCs, embryonic stem cells; ExE, extraembryonic ectoderm; FACS, fluorescence-activated cell sorting; GlyTs, trophoblast glycogen cells; GSEA, gene set enrichment analysis; ICM, inner cell mass; JZ, junctional zone; Lab, labyrinth; LaTPs, labyrinth trophoblast progenitors; MEFs, mouse embryonic fibroblasts; Mv, microvilli; PRL, prolactin; P-TGCs, parietal trophoblast giant cells; SpA-TGCs, spiral artery-associated trophoblast giant cells; SpTs, spongiotrophoblasts; S-TGCs, sinusoidal trophoblast giant cells; SynTII, syncytiotrophoblast layer II; SynTIs, syncytiotrophoblasts layer I; TBLCs, totipotent blastomere-like cells; TE, trophectoderm; TE3.5, TE cells of E3.5 embryos; TELCs, trophectoderm-like cells; TELSCs, trophectoderm-like stem cells; TEM, transmission electron microscopy; TESCs, trophectoderm stem cells; TGCs, trophoblast giant cells; TOs, trophoblast organoids; TSCs, trophoblast stem cells; TSSs, transcription start sites; WGBS, whole-genome bisulfite sequencing.
