# Supplementary material for: Capturing trophectoderm-like stem cells enables step-wisely remodeling of placental development
Source: Protein Cell. 2025 Nov 10;17(4):339–56. doi: 10.1093/procel/pwaf098 (PMC13107561; doi:10.1093/procel/pwaf098)
Supplement: pwaf098_Supplementary_Data [file pwaf098_supplementary_data.zip › PAC-25532-DP-Supplementary materials(1).docx]

**
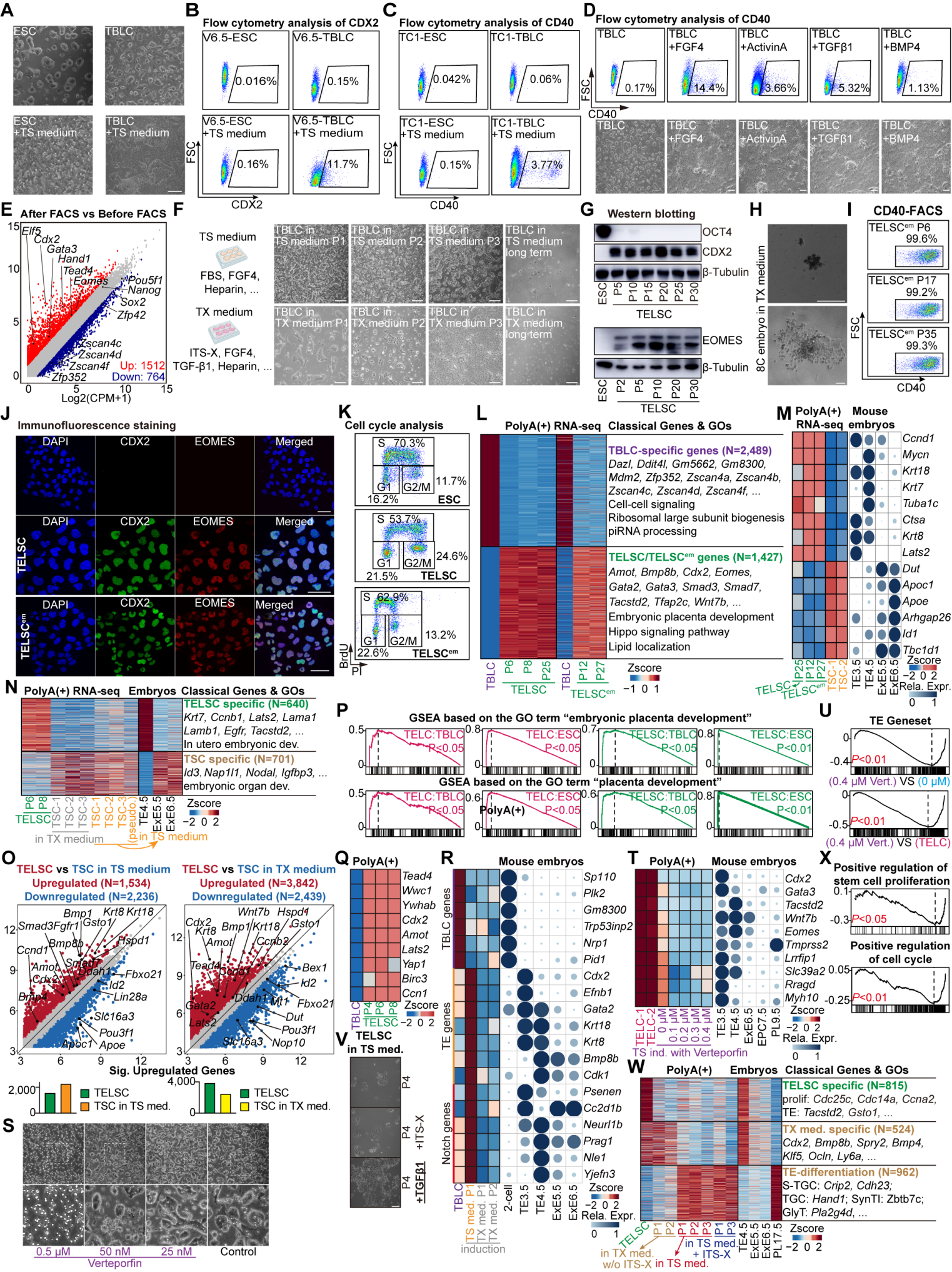
**

**Figure S1. Capturing trophectoderm-like stem cells (TELSCs) with pre-implantation E4.5 TE features using a “two-step” culture system, related to Figure 1.**

(A) The morphology of ESCs, TBLCs and ESCs, TBLCs in TS medium after 3 days of induction. Scale bars, 250 μm.

(B) FACS analysis of the percentage of CDX2^+^ cells from ESCs and TBLCs, as well as ESCs and TBLCs cultured in TS medium, using V6.5 cell line.

(C) FACS analysis of the percentage of CD40^+^ cells from ESCs and TBLCs, as well as ESCs and TBLCs cultured in TS medium, using TC1 cell line.

(D) FACS analysis of the percentage of CD40^+^ TELCs obtained from the TBLCs after induction with different molecules, including FGF4, Activin A, TGFβ1 and BMP4. The corresponding cell morphology is displayed in the lower panel.

(E) Scatterplots displaying the transcriptome comparison of TELCs before and after CD40-based FACS using RNA-seq. Upregulated (FC>2) and downregulated (FC<0.5) genes are shown in red and blue, respectively.

(F) The morphology of TBLCs of different passages and long-term culture in TX and TS medium, also the morphology of TBLCs after CD40 FACS after induction. Scale bars, 250 μm.

(G) Western blotting was used to detect OCT4, CDX2 and EOMES in TELSCs from different passages. β-Tubulin was used as a loading control.

(H) The morphology 8C embryos cultured in TX medium. Scale bars, 250 μm.

(I) FACS analysis of the percentage of CD40^+^ cells in TELSC^em^s at different passages.

(J) Immunofluorescence staining of TFAP2C and PEG10 in TBLCs, TELSCs and TELSC^em^s. Scale bars, 50 μm.

(K) Cell cycle analysis of ESCs, TELSCs and TELSC^em^s.

(L) Heatmap indicating the relative expression of TBLCs, TELSCs and TELSC^em^s. The representative genes and enrichment of GO terms of these genes is shown.

(M) Heatmap indicating the relative expression of characteristic genes in TELSCs, TELSC^em^s and TSCs. Bubble chart showing the relative expression of these genes in mouse embryos.

(N) Heatmap indicating the relative expression of characteristic genes in TELSCs, TSCs cultured in TX medium and TSCs cultured in TS medium. Heatmap on the right demonstrating the expression of each cluster in mouse embryos. The representative genes and enrichment of GO terms of these genes is shown.

(O) The scatter plot displays differentially expressed genes between TELSCs and TSCs cultured in various media. The bar graph summarizes the number of differentially expressed genes identified under each comparison condition.

(P) GSEA analysis of ESCs, TBLCs, TELCs and TELSCs based on “embryonic placenta development” and “placenta development” geneset.

(Q) Heatmap indicating the differentially expressed genes in Hippo pathway of TELSCs and TBLCs.

(R) Heatmap indicating the relative expression of characteristic genes in TELSCs, TSCs cultured in TX medium and TSCs cultured in TS medium. Bubble chart showing the relative expression of these genes in mouse embryos.

(S) Phase contrast images of TBLCs cultured in TS medium for 24h supplemented with Verteporfin at the indicated concentration. Scale bars, 100 µm.

(T) Heatmap indicating the differentially expressed genes of TELCs and TBLCs induction in TS medium plus verteporfin. Bubble chart showing the relative expression of these genes in mouse embryos.

(U) GSEA analysis of TELCs, TBLCs induction in TS medium and in TS medium plus verteporfin based on TE geneset.

(V) The morphology of TELSCs cultured in TS medium, TS medium plus ITS-X and TS medium plus TGFβ1.

(W) Heatmap indicating the differentially expressed genes of TELSCs, TBLCs induction in TX medium withdraw ITS-X, in TS medium and in TS medium plus ITS-X. Heatmap on the right demonstrating the expression of each cluster in mouse embryos. The representative genes and enrichment of GO terms of these genes is shown.

(X) GSEA analysis of TBLCs induction in TX medium withdraw ITS-X and in TX medium based on “Positive regulation of stem cell proliferation” and “Positive regulation of cell cycle” geneset.

##
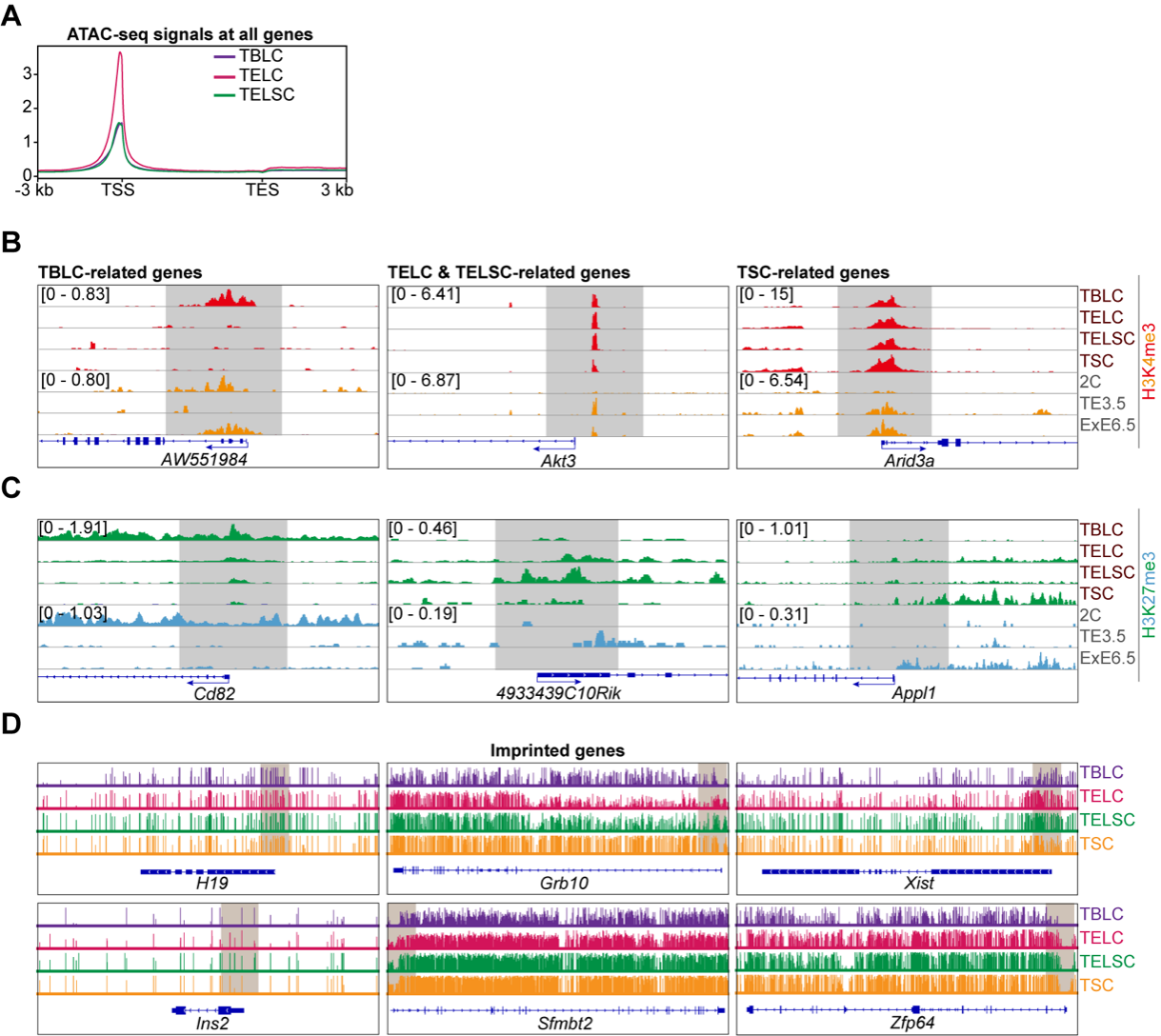


**Figure S2. The unique epigenomic features of TELCs and TELSCs, related to Figure 2.**

(A) Average ATAC-seq signals at all genes in TBLCs, TELCs and TELSCs.

(B) IGV browser view displaying H3K4me3 signals of specific genes in TBLCs, TELCs, TELSCs and TSCs, and mouse embryos (Liu et al., 2016; Andrews et al., 2023).

(C) IGV browser view displaying H3K27me3 signals of specific genes in TBLCs, TELCs, TELSCs and TSCs, and mouse embryos (Liu et al., 2016; Andrews et al., 2023).

(D) IGV browser view displaying DNA methylation patterns of imprinting genes in TBLCs, TELCs, TELSCs and TSCs.

##
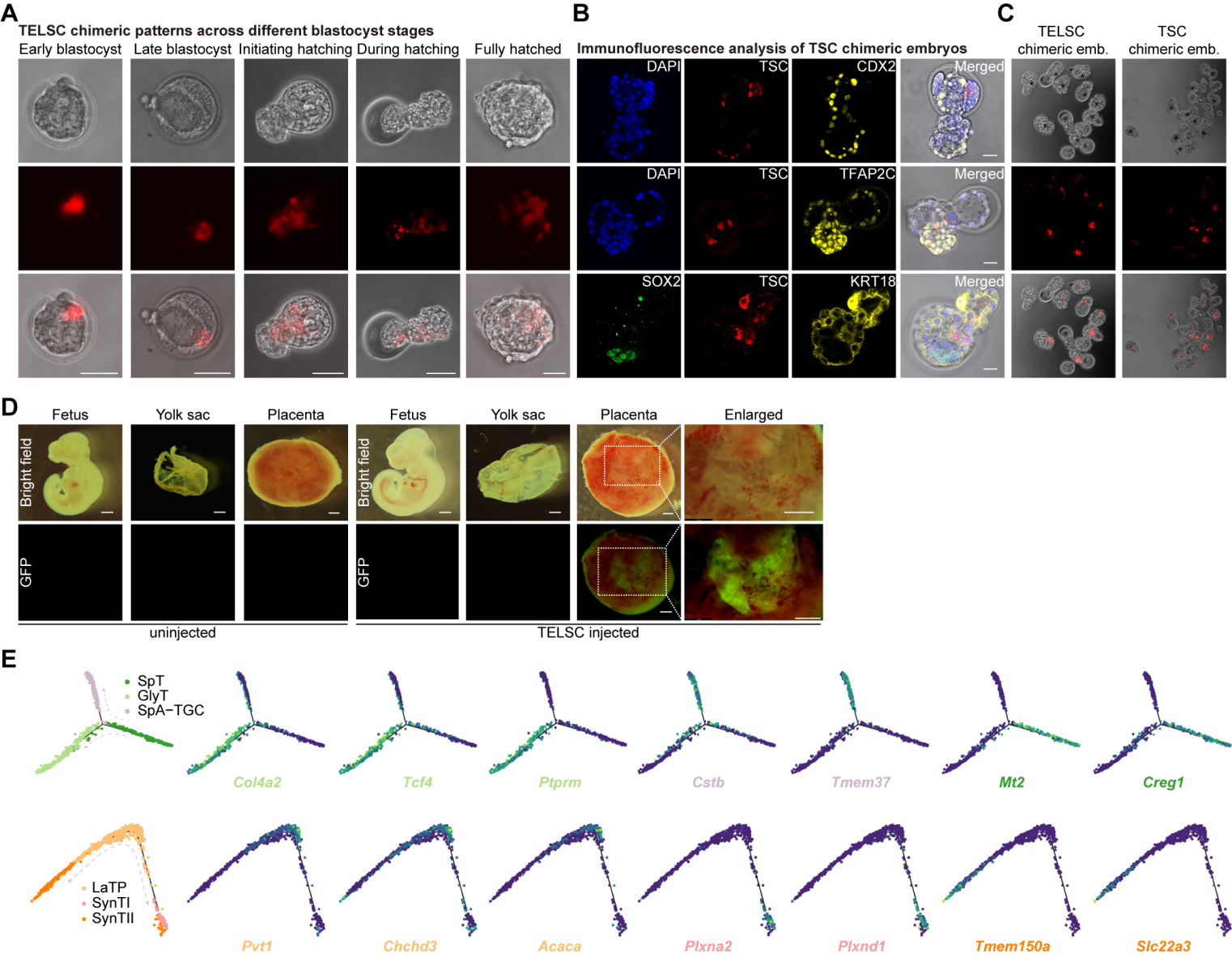


**Figure S3. TELSCs exhibit robust *in vivo* developmental potential and full trophoblast lineage contribution, related to Figure 3.**

(A) Representative images of chimeric embryos injected mCherry-labeled TELSCs. TELSCs can cooperate into trophectoderm across blastocyst developmental stages: early blastocyst, late blastocyst, hatching-initiation blastocyst, hatching-progress blastocyst, and fully hatched (zona pellucida-free) blastocyst. Scale bar, 50 μm.

(B) Representative immunostaining of chimeric blastocysts injected with mCherry-labeled TSCs. CDX2, TFAP2C and KRT18: TE-speciﬁc markers; SOX2: ICM-specific marker. Scale bar, 20 µm.

(C) Images of chimeric embryos injected with mCherry-labeled TELSCs or TSCs. Asterisk, TELSCs or TSCs contribute to TE and form chimeric embryos.

(D) Images of chimeric conceptuses derived from 8-cell embryos injected with donor EGFP-labeled TELSCs with an uninjected conceptus as control. Scale bar, 1 mm.

(E) Monocle2 analysis of lineage-specific gene expression of TELSC-chimeric placenta across single-cell differentiation trajectories.


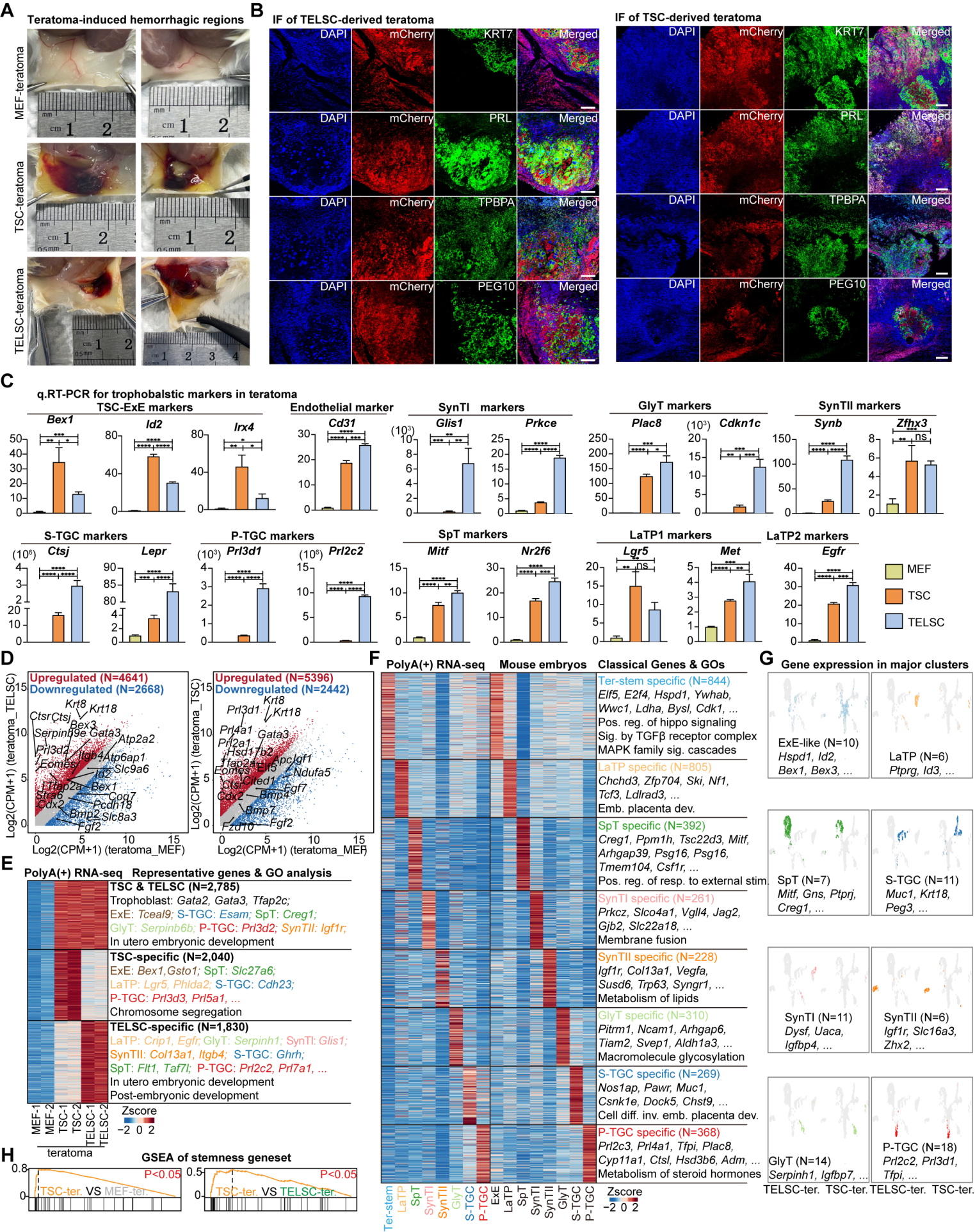


**Figure S4. TELSCs exhibit enhanced *in vivo* trophoblast differentiation potential compared to TSCs in teratoma assays, related to Figure 4.**

(A) TSCs, TELSCs or control MEFs were injected subcutaneously into both flanks of NOG mice. Lesions were analyzed 10 days after injection. Replication of (Fig. 4B), demonstrating the reproducibility of the results.

(B) Immunofluorescence analysis of teratoma tissues derived from TELSCs and TSCs. Scale bars, 200 mm.

(C) qRT-PCR analysis of the relative expression of the trophoblast lineage marker of teratoma derived from MEFs, TSCs and TELSCs. Data were normalized to GAPDH. Mean ± SD; n = 3 technical replicates and statistically significant differences (Student's t test) are indicated with asterisks.

(D) Scatterplots displaying the transcriptome of teratoma derived from MEFs, TSCs and TELSCs using RNA-seq. Upregulated (FC > 2) and downregulated (FC < 0.5) genes are shown in red and blue, respectively.

(E) Heatmaps of the relative expression of commonly and specifically upregulated genes in teratoma derived from TSCs, TELSCs compared with teratoma derived from MEFs. The representative genes and enrichment of GO terms of these genes is shown.

(F) The heatmap displays differentially expressed genes across clusters identified in TELSC-derived teratomas. The expression profiles of these genes are also shown in corresponding stages of *in vivo* mouse placental development.

(G) UMAP visualizations showing the expression of the marker genes in major clusters in teratoma derived from TSCs, TELSCs.

(H) GSEA analysis of teratoma derived from MEFs, TSCs and TELSCs based on stemness geneset.


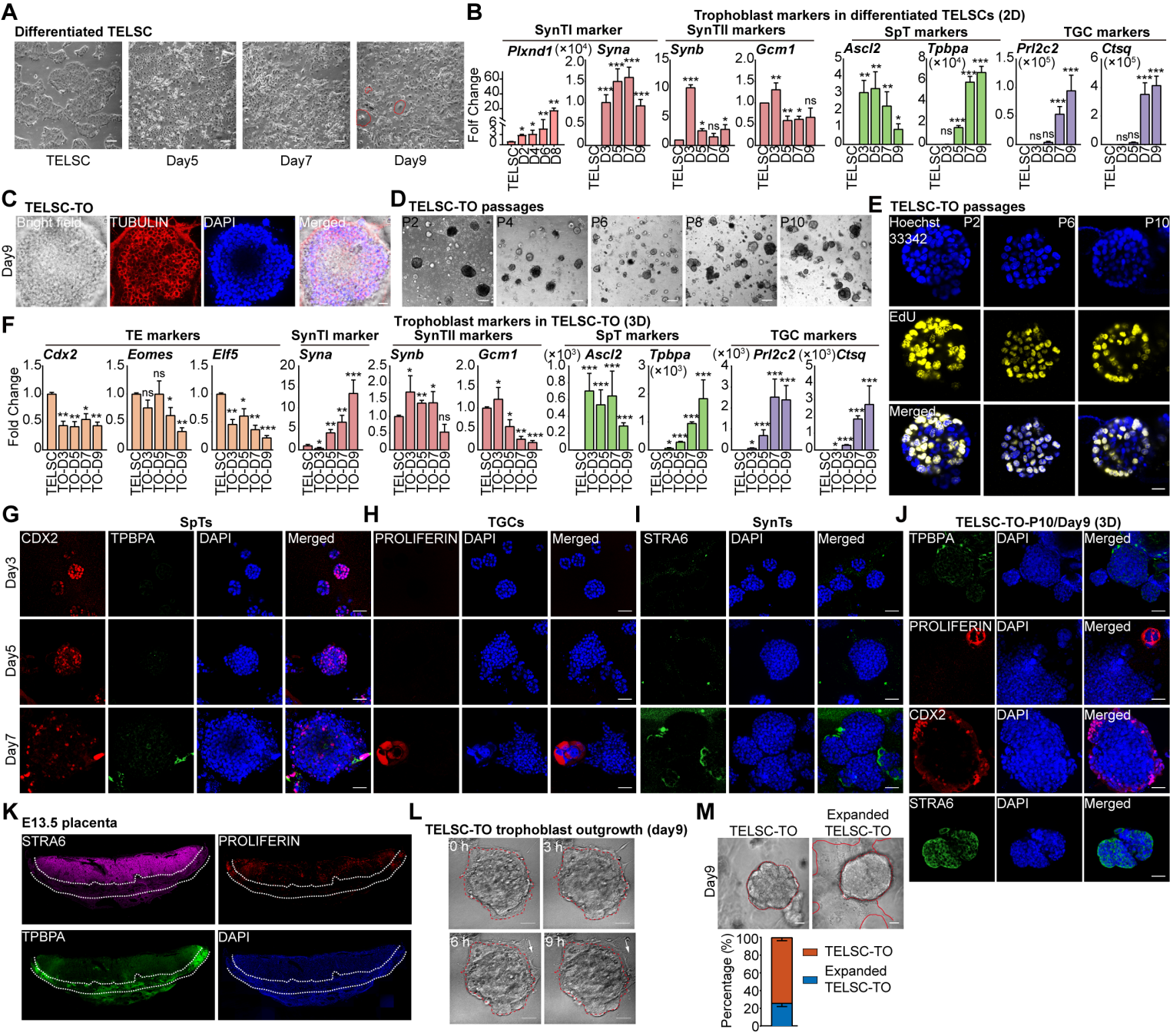


**Figure S5. TELSCs, but not TSCs, can efficiently generate trophoblast organoids with mature trophoblast lineages and self-renewal ability, related to Figure 5.**

(A) Morphology of long-term cultured TELSCs and differentiated TELSCs in 2D culture condition. Scale bars, 25 μm.

(B) qRT-PCR analysis of the relative expression of different trophoblast markers in TELSCs and differentiated TELSCs in 2D culture condition at different time points. Data were normalized to GAPDH. Mean ± SD; n = 3 technical replicates. *: p value < 0.05, **: p value < 0.01,***: p value < 0.001, unpaired Student’s t test. ns: not significant.

(C) The structure of TELSC-TO at day 9. Immunofluorescence staining of TUBULIN to show the cytoskeletal structure of TELSC-TO. Scale bars, 25 μm.

(D) Morphology of long-term cultured TELSC-TO. Scale bars, 25 μm.

(E) EdU staining of long-term cultured TELSC-TO at different passages. Scale bars, 25 μm.

(F) qRT-PCR analysis of the relative expression of different trophoblast markers during TELSC-TO formation: Cdx2, Eomes and Elf5 for TE; Syna for SynTI; Synb and Gcm1 for SynTII; Ascl2 and Tpbpa for SpT; Prl2c2 and Ctsq for TGC. Data were normalized to GAPDH. Mean ± SD; n = 3 technical replicates. *: p value < 0.05, **: p value < 0.01,***: p value < 0.001, unpaired Student’s t test. ns: not significant.

(G-I) Immunofluorescence staining of trophoblast organoids. TPBPA for SpT, PROLIFERIN for TGC and STRA6 for SynTI. Scale bars, 50 μm.

(J) Immunofluorescence staining of trophoblast organoids in P10. TPBPA for SpT, PROLIFERIN for TGC and STRA6 for SynTI. Scale bars, 50 μm.

(K) Immunohistochemistry analysis of placenta sections from E13.5 conceptuses. The placenta was stained with PROLIFERIN, TPBPA and STRA6. Scale bars, 1000 μm.

(L) Long-term morphological changes in TELSC-TO. The arrowheads represent the outgrowth of TELSC-TO. Scale bars, 25 μm.

(M) Morphology of expanded TELSC-TO at day 9 and corresponding statistics. Scale bars, 25 μm.


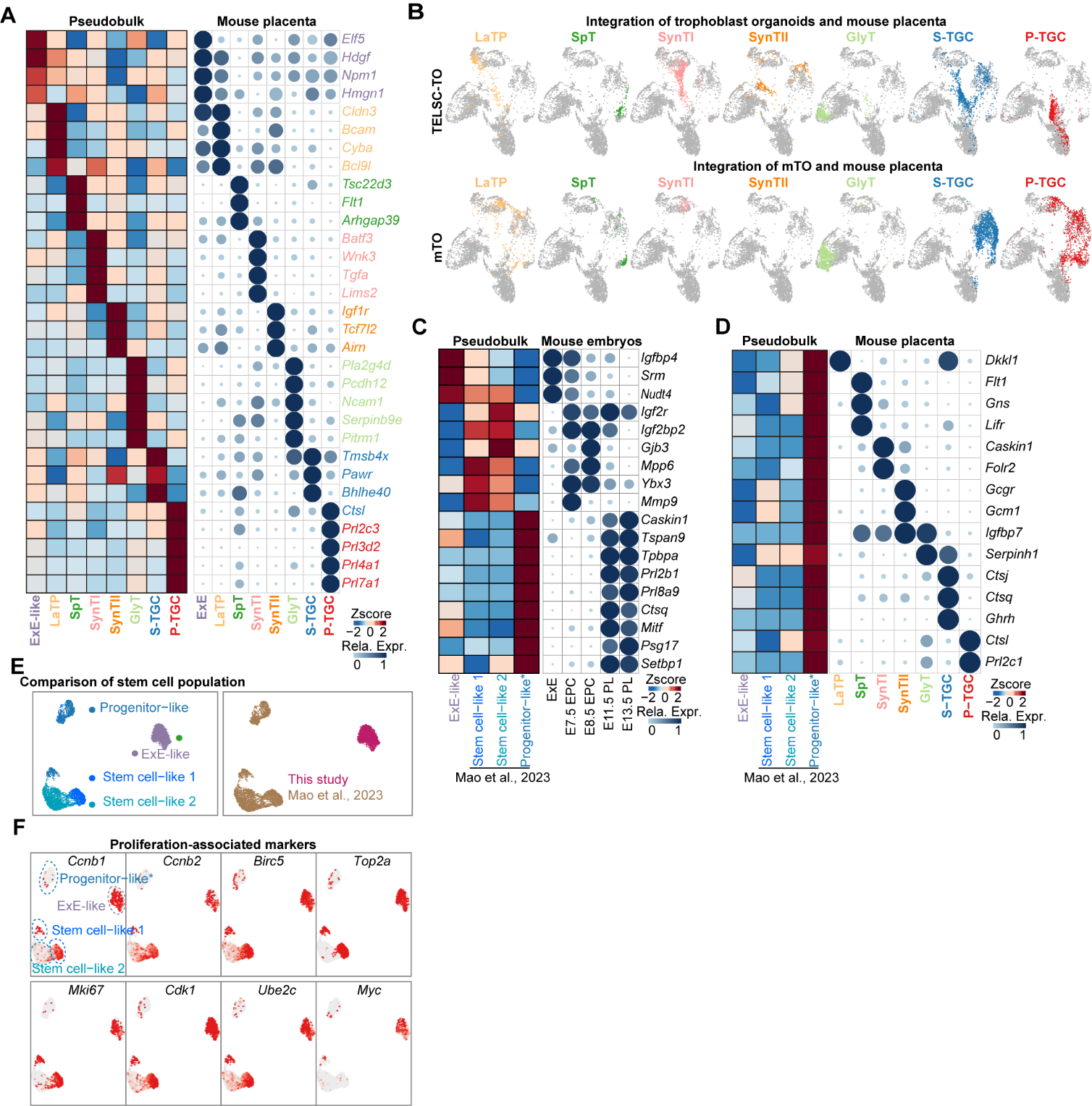


**Figure S6. Newly identified ExE-like progenitors enable coupled self-renewal and differentiation abilities of TELSC-derived organoids, related to Figure 6.**

(A) Heatmap and bubble chart displaying marker gene expression in trophoblast cell types in TELSC-TO and mouse embryos.

(B) UMAP visualizations showing the integration of TELSC-TO, TO reported by (Mao et al., 2023) and mouse placenta, the gray points represent mouse embryonic data.

(C) Heatmap on the left demonstrating the DEGs between ExE-like from TELSC-TO and stem cell population from (Mao et al., 2023) Bubble chart showing the relative expression of these genes in mouse embryos.

(D) Heatmap and bubble chart displaying marker gene expression in ExE-like, stem cell population from (Mao et al., 2023) and the reported mouse trophoblast cell types.

(E) The integration of the scRNA-seq results of stem cell population from TELSC-TO (ExE-like) and from TO reported by (Mao et al., 2023).

(F) UMAP visualizations showing the expression of the proliferation-associated marker genes in stem cell population.


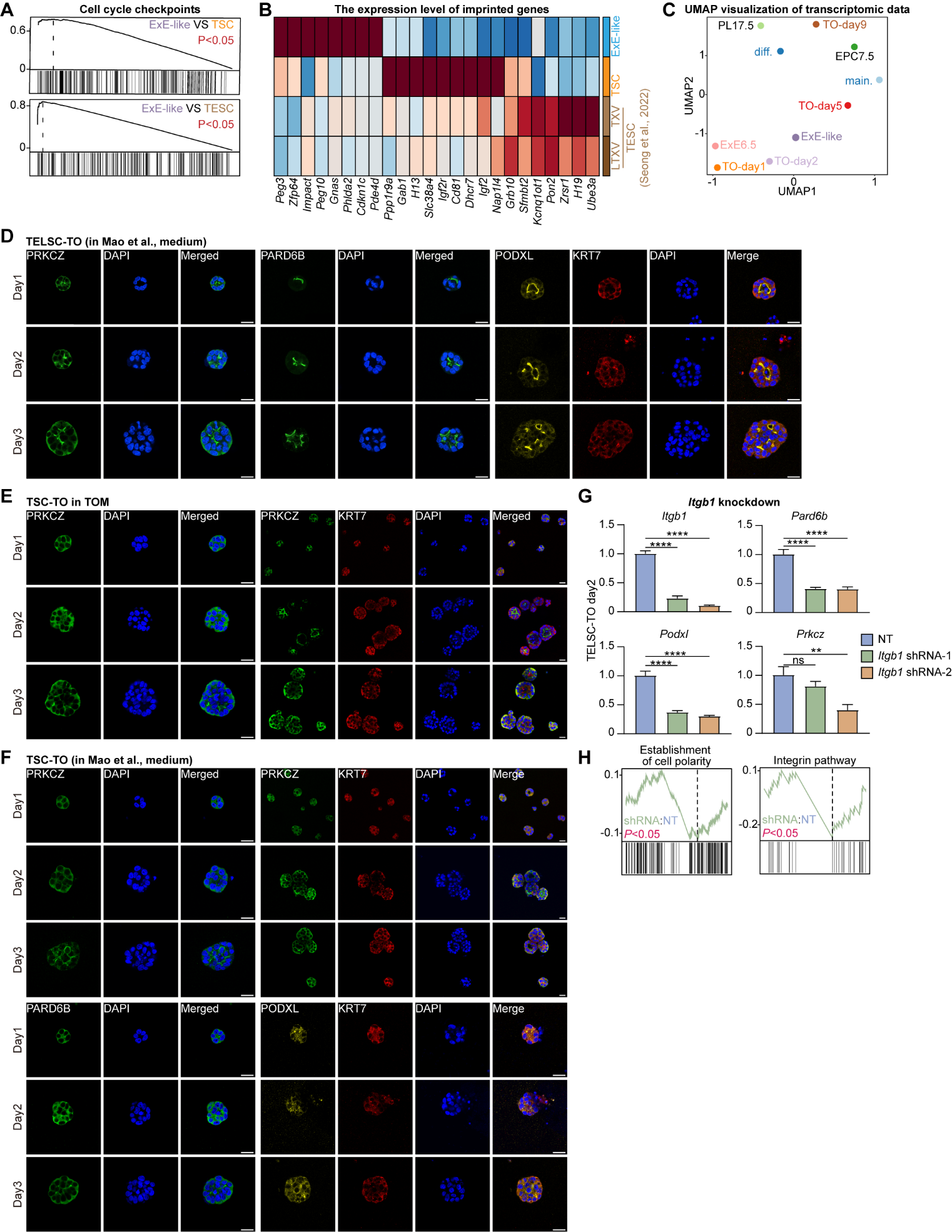


**Figure S7. The rosette structure, relying on ITGB1, is required for ExE-like progenitor induction and TELSC-derived TO formation, related to Figure 7.**

(A) GSEA analysis of ExE-like, TSCs and TESCs based on “Cell cycle checkpoints” geneset.

(B) Heatmap comparing placenta-associated imprinted gene expression across ExE-like, TSCs, and TESCs.

(C) UMAP analysis of bulk RNA-seq of TELSC-TO, pseudo-bulk of mTO from (Mao et al., 2023) and also mouse embryos.

(D) Immunofluorescence staining of PARD6B, PODXL, PRKCZ and KRT7 in TELSC-TO from day 1 to day 3. Scale bars, 25 μm.

(E-F) Immunofluorescence staining of PARD6B, PODXL, PRKCZ and KRT7 in TSC-TO from day 1 to day 3. Scale bars, 25 μm.

(G) qRT-PCR analysis of the relative expression of *Itgb1*, *Pard6b*, *Podxl* and *Prkcz* after transfected with *Itgb1* shRNA.

(H) GSEA analysis of TELSC-TO and TELSC-TO transfected with *Itgb1* shRNA based on “Establishment of cell polarity” and “Integrin pathway” geneset.

# EXPERIMENTAL MODEL AND SUBJECT DETAILS

**KEY RESOURCES TABLE**

| **REAGENT or RESOURCE** | **SOURCE** | **IDENTIFIER** |
| --- | --- | --- |
| Antibodies | | |
| Fluorescence-conjugated primary antibodies PE-CD40 | BioLegend | Cat#124605 |
| Cdx2 Antibody | Abcam | Cat# ab76541 |
| Eomes Antibody | Abcam | Cat# ab216870 |
| β-Tubulin Antibody | Cell Signaling Technology | Cat# 2146 |
| Cdx2 Antibody | BioGenex | Cat# MU392A-UC |
| Hand1 Antibody | Santa Cruz | Cat# sc-390376 |
| Tpbpa Antibody | Abcam | Cat# ab104401 |
| Stra6 Antibody | R&D Systems | Cat# ABN1662 |
| Krt7 Antibody | Abcam | Cat# ab181598 |
| Igf1r Antibody | Abcam | Cat# ab182408 |
| Proliferin Antibody | Santa Cruz | Cat#sc-271891 |
| H3K4me3 Antibody | Abcam | Cat# ab6002 |
| H3K27me3 Antibody | Abcam | Cat# ab213224 |
| E-cadherin Antibody | Santa Cruz | Cat# sc-8426 |
| Anti-BrdU Antibody | Abcam | Cat#ab6326 |
| Goat anti-Rabbit IgG (H+L) Highly Cross-Adsorbed Secondary Antibody, Alexa Fluor 647 | ThermoFisher | Cat#A32733 |
| Goat anti-Mouse IgG (H+L) Cross-Adsorbed Secondary Antibody, Alexa Fluor 488 | ThermoFisher | Cat#A11001 |
| Goat anti-Rabbit IgG (H+L) Cross-Adsorbed Secondary Antibody, Alexa Fluor 488 | ThermoFisher | Cat# A21206 |
| Goat anti-Mouse IgG (H+L) Cross-Adsorbed Secondary Antibody, Alexa Fluor 594 | ThermoFisher | Cat# A11005 |
| HRP Goat Anti-Mouse IgG (H+L) | Biodragon | Cat# BF03001 |
| HRP Goat Anti-Rabbit IgG (H+L) | Biodragon | Cat# BF03008 |
| Bacterial and Virus Strains | | |
| N/A | N/A | N/A |
| Biological Samples |  |  |
| N/A | N/A | N/A |
| Chemicals, Peptides, and Recombinant Proteins | | |
| DMEM/F12 | GIBCO | Cat#11330032 |
| Advanced DMEM/F12 | GIBCO | Cat#12634010 |
| DMEM | BI | Cat#06-1055-57-1ACS |
| RPMI 1640 | GIBCO | Cat#11875119 |
| Fetal Bovine Serum | Excell | Cat#FSP500 |
| Mouse Leukemia Inhibitory Factor (LIF) | Millipore | Cat#ESG1107 |
| TRYPLE EXPRESS W/PHENOL RED | GIBCO | Cat#12605028 |
| Non-Essential Amino Acids Solution (NEAA) | GIBCO | Cat#11140050 |
| L-Glutamine | GIBCO | Cat#25030081 |
| Sodium Pyruvate | GIBCO | Cat#11360070 |
| GlutaMax | GIBCO | Cat# 35050061 |
| Penicillin-Streptomycin | GIBCO | Cat#15140163 |
| Trypsin-EDTA | GIBCO | Cat#15400054 |
| B-27 Supplement | GIBCO | Cat#12587010 |
| N2 Supplement | GIBCO | Cat#17502048 |
| 2-Mercaptoethanol | ThermoFisher | Cat#60-24-2 |
| Gelatin from porcine skin | Sigma | Cat# G1890 |
| DPBS | GIBCO | Cat#14190250 |
| Heparin | STEMCELL | Cat#7980 |
| Human recombinant FGF4 | MCE | Cat# HY-P7014 |
| Mouse recombinant TGF-β1 | PeproTech | Cat#100-21-10 |
| Recombinant EGF | PeproTech | Cat#315-09-100 |
| Recombinant R-spondin-1 | MCE | Cat# HY-P7114 |
| Recombinant FGF-2 | PeproTech | Cat#450-33-50 |
| Recombinant HGF | PeproTech | Cat#315-23-20 |
| Prostaglandin E2 | Sigma | Cat#P0409 |
| Growth-factor-reduced Matrigel | Corning | Cat#354230 |
| Growth-factor-reduced Matrigel, Phenol Red-free | Corning | Cat#354231 |
| ITS-X | GIBCO | Cat#51500056 |
| CHIR-99021 | Selleck | Cat#S2924 |
| A-83-01 | Tocris | Cat#2939 |
| Y-27632 2HCL | Selleck | Cat#S1049 |
| Pladienolide B | Tocris | Cat#6070/500U |
| N-Acetyl-L-cysteine | Sigma | Cat# A9165 |
| L-ascorbic acid | MCE | Cat#HY-B0166 |
| Bovine Serum Albumin | Sigma | Cat#B2064 |
| Triton X-100 | Sigma | Cat#X100-500ML |
| Tween20 | Sigma | Cat#P2287-500ML |
| DTT | Sigma | Cat#D0632 |
| Q5 HF Master Mix | NEB | Cat#M0492S |
| Proteinase K | NEB | Cat#P8107S |
| PMSF | Beyotime | Cat#ST506 |
| TRIzol^TM^ Reagent | Invitrogen | Cat#15596026 |
| UltraPure^TM^ DNase/RNase-Free Distilled Water | Invitrogen | Cat#10977015 |
| NuPAGE^TM^ LDS Sample Buffer (4X) | ThermoFisher | Cat#NP0007 |
| 4% Paraformaldehyde Fix Solution (PFA) | BBI Life Science | Cat#E672002-0100 |
| DAPI solution | Solarbio | Cat# C0060 |
| Polybrene | Solarbio | Cat#H8761 |
| DNase I | Promega | Cat#M6101 |
| 0.4% trypan blue staining solution | Coolaber | Cat#SL7120 |
| Critical Commercial Assays | | |
| VAHTS Universal V8 RNA-seq Library Prep Kit for Illumina | Vazyme | Cat#NR605 |
| EpiArt DNA Methylation Bisulfite Kit | Vazyme | Cat# EM101-01 |
| Epitect Bisulfite Kit | QIANGEN | Cat#59104 |
| EpiArt DNA Methylation Library Kit for Illumina V3 | Vazyme | Cat#NE103-01 |
| ZymoTaq PreMix | Zymo | Cat# E2004 |
| Hyperactive Universal CUT&Tag Assay Kit for Illumina | Vazyme | Cat#TD903-01 |
| Hyperactive ATAC-Seq Library Prep Kit for Illumina | Vazyme | Cat#TD711-01 |
| VAHTS mRNA Capture Beads | Vazyme | Cat#N401 |
| HiScript Ⅱ RT SuperMix for qPCR | Vazyme | Cat#R223 |
| VAHTS DNA Clean Beads | Vazyme | Cat#N411 |
| VAHTS RNA Adapters | Vazyme | Cat#N810 |
| VAHTS RNA Adapters ser1 for Illumina | Vazyme | Cat#N803 |
| TruePrep Index Kit V4 | Vazyme | Cat#TD204 |
| TruePrep DNA Library Prep Kit V2 | Vazyme | Cat#TD501 |
| P3 Primary Cell 4D-Nucleofector X Kit | Lonza | Cat#V4XP-3024 |
| TIANamp Genomic DNA Kit | TIANGEN | Cat#DP304 |
| 2X M5 HiPer Realtime PCR Super mix | Mei5 Biotech | Cat#MF013 |
| SurePAGE™, Bis-Tris, 10x8, 4-12%, 15 wells | Genscript | Cat#M00654 |
| Immobilon-P PVDF Membrane, 0.45 μm | Millipore | Cat#IPVH07850 |
| Chromium Next GEM Single Cell 3ʹGEM Kit v3.1 | 10x Genomics | Cat#PN-1000127 |
| Chromium Next GEM Chip G | 10x Genomics | Cat#PN-1000120 |
| Chromium i7 Multiplex Kit | 10x Genomics | Cat#PN-120262 |
| SPRIselect Reagent Kit | Beckman | Cat#B23318 |
| FastDigest BbsI (BpiI) | ThermoFisher | Cat# FD1014 |
| T4 DNA ligase | NEB | Cat# M0202L |
| 1.1 × T3 Super PCR Mix | TSINGKE | Cat# T-TSE030 |
| SuperScript IV RT kit | Invitrogen | Cat#18090010 |
| Lipofectamine™ 3000 | Invitrogen | Cat#11668019 |
| Deposited Data | | |
| RNA-seq | This paper | GSE251985 |
| ATAC-seq & ChIP-seq | This paper | GSE251985 |
| WGBS | This paper | GSE251985 |
| scRNA-seq | This paper | GSE251985 |
| scRNA-seq of mouse placentas | (Jiang et al., 2023) | GSE156125 |
| scRNA-seq of mouse embryos | (Nowotschin et al., 2019) | GSE123046 |
| scRNA-seq of mouse embryos | (Cheng et al., 2019) | GSE109071 |
| RNA-seq of mouse embryos | (Wang et al., 2018) | GSE98150 |
| RNA-seq of mouse trophectoderm | (Sun et al., 2021) | GSE163379 |
| RNA-seq of trophectoderm stem cells | (Seong et al., 2022) | GSE200960 |
| RNA-seq of mouse ectoplacental cone | (Tuteja et al., 2016) | GSE65808 |
| RNA-seq of mouse placentas | (Chu et al., 2019) | GSE112755 |
| RNA-seq & WGBS & ATAC-seq of TBLCs | (Shen et al., 2021) | GSE168728 |
| WGBS of mouse trophectoderm | (Wang et al., 2018) | GSE98151 |
| WGBS of TSCs | (Weigert et al., 2023) | GSE196977 |
| WGBS of mouse placentas | (Hon et al., 2013) | GSE42836 |
| Experimental Models: Cell Lines | | |
| Mouse cell line: V6.5 ES cells | N/A | N/A |
| Mouse cell line: TC1 ES cells | Laboratory of Frederick Alt | N/A |
| Mouse cell line: TS cells | Laboratory of Yanling Wang | N/A |
| Recombinant DNA | | |
| psPAX2 | N/A | N/A |
| pMD2.G | N/A | N/A |
| pSIN-EGFP | N/A | N/A |
| Oligonucleotides | | |
| Primers for qPCR, see method details | This study | N/A |
| Software and Algorithms | | |
| R version 3.6.0 | The R Foundation for Statistical Computing | <https://www.r-project.org/> |
| Cell Ranger 3.0.2 | 10x Genomics | <https://www.10xgenomics.com/> |
| Seurat 3.2.3 | (Stuart et al., 2019) | <https://satijalab.org/seurat/> |
| Limma 3.42.2 | (Ritchie et al., 2015) | https://bioconductor.org/packages/release/bioc/html/limma.html |
| GSVA 1.34.0 | (Hänzelmann et al., 2013) | https://www.bioconductor.org/packages/release/bioc/html/GSVA.html |
| ClusterProfiler 3.14.3 | (Yu et al., 2012) | <https://bioconductor.org/packages/release/bioc/html/clusterProfiler.html> |
| MethylKit 1.12.0 | (Akalin et al., 2012) | https://bioconductor.org/packages/release/bioc/html/methylKit.html |
| DSS 2.34.0 | (Wu et al., 2013) | https://bioconductor.org/packages/release/bioc/html/DSS.html |
| BiomaRt 2.40.5 | (Durinck et al., 2005) | https://bioconductor.org/packages/release/bioc/html/biomaRt.html |
| Trimmomatic 0.39 | (Bolger et al., 2014) | <http://www.usadellab.org/cms/?page=trimmomatic> |
| HISAT2 2.1.0 | (Kim et al., 2019) | <https://daehwankimlab.github.io/hisat2/> |
| FeatureCounts 1.6.4 | (Liao et al., 2014) | <http://subread.sourceforge.net/> |
| SNPsplit 0.4.0 | (Krueger and Andrews, 2016) | https://github.com/FelixKrueger/SNPsplit |
| ATAC-seq pipline | (Koh et al., 2016) | https://github.com/ENCODE-DCC/atac-seq-pipeline |
| Bowtie2 2.4.4 | (Langmead et al., 2019) | http://bowtie-bio.sourceforge.net/bowtie2/manual.shtml |
| Samtools 1.9 | (Li et al., 2009) | https://github.com/samtools/samtools |
| MACS2 2.1.4 | (Zhang et al., 2008) | https://hbctraining.github.io/Intro-to-ChIPseq/lessons/05_peak_calling_macs.html |
| Other | | |
| N/A | N/A | N/A |

**RESOURCE AVAILABILITY**

**Lead contact**

Further queries and reagent requests may be directed and will be fulfilled by the lead contact, Peng Du ([pengdu@pku.edu.cn](mailto:pengdu@pku.edu.cn)).

**Data and code availability**

The RNA-seq, scRNAseq, WGBS, ATAC-seq and ChIP-seq data generated in this study are available at China National Center for Bioinformation, with accession number PRJCA049383. All samples generated during this study have been summarized in **Table S7**.

**EXPERIMENTAL MODEL AND SUBJECT DETAILS**

**METHOD DETAILS**

**Cell culture**

All cell lines were cultured under 20% O_2_ and 5% CO_2_ at 37°C, and the medium was refreshed daily. Cells were passaged every 2-3 days with 0.25% trypsin-EDTA (GIBCO, 15400054). All ESCs (V6.5, TC1, M3, female ESCs and reporter cell lines) were cultured on mitomycin-treated mouse embryonic fibroblast (MEF) feeder layers or 0.1% gelatin (Sigma, G1890), in serum/LIF medium composed of DMEM (GIBCO, 11965092) supplemented with 20% fetal bovine serum (FBS) (ExCell, FCS500), 1% L-glutamine (GIBCO, 25030081), 1% penicillin-streptomycin (GIBCO, 15140163), 1% non-essential amino acids (NEAA) (GIBCO, 11140050), 1% sodium pyruvate (GIBCO, 11360070), 50 mM 2-mercaptoethanol (ThermoFisher, 60-24-2) and 1000 U/mL mouse leukemia inhibitory factor (mLIF) (Millipore, ESG1107). Feeder cells were seeded (5×10^4^ cells per cm^2^) onto 0.1% gelatin-coated dishes at least 12 hours before seeding ESCs. Feeder plates were used within 3 days.

All TSLs were cultured on Matrigel-coated plates, in 30% TS medium (RPMI 1640 (GIBCO, 11875119), 20% FBS, 1% GlutaMax (GIBCO, 35050061), 1% penicillin-streptomycin (GIBCO, 15140163), 1% sodium pyruvate (GIBCO, 11360070)) and 70% MEF-conditioned TS medium supplemented with 25 ng/mL human recombinant FGF4 (MCE, HY-P7014) and 1 μg/mL heparin (STEMCELL, 7980). For longer culture, the medium was changed to serum-free TX medium (Garreta et al., 2021; Kim et al., 2020; Rossi et al., 2018): F12/DMEM (GIBCO, 11330057), 64 mg/L L-ascorbic acid-2-phosphate magnesium, 1% penicillin-streptomycin, 1% sodium pyruvate, and 2% Insulin-Transferrin-Selenium-X (GIBCO, 51500056), supplemented with 25 ng/mL human recombinant FGF4, 1 μg/mL heparin, and 2 ng/mL mouse recombinant TGF-β1 (PeproTech, 100-21-10). TSLs were passaged every three days by incubation in TrypLE (GIBCO, 12605028) for 3 minutes, and the enzyme was inactivated by the addition of TS medium.

To generate TBLCs, ESCs were cultured in SLP medium (serum/LIF medium supplemented with 2.5 nM PlaB (Tocris, 6070) or 10 nM plaB (Kubaczka et al., 2014). For the first passage, cells were plated in serum/LIF medium, and the medium was changed to SLP medium the following day. For cryopreservation, cell pellets were resuspended in FBS/10% DMSO and stored at -80℃ or in liquid nitrogen.

**Derivation of TELSC^em^ line from 8-cell embryos**

Using a microscope, the zona pellucida of at least 40 eight-cell mouse embryos was digested with Tyrode's solution at room temperature for approximately 5 minutes. Following digestion, embryos were transferred via mouth pipette to a gelatin-coated 48-well plate and cultured in TS medium. Initially, cell proliferation was slow, with visible cell aggregates appearing after approximately one week. These cells were then transitioned into TX medium for extended culture over at least five passages. The establishment of the TELSC^em^ line was confirmed through RNA sequencing and immunofluorescence staining.

**Teratoma formation assay**

MEFs, TSCs or TELSCs were digested into single cells, and approximately 5×10^6^ cells were collected and re-suspended in 100 μl of Matrigel. Next, the cells were subcutaneously injected into each side of 6- to 8-week-old immunodeficient NOG female mice. After growth for 1-2 weeks on average (day 10), all mice were euthanized via cervical dislocation and teratomas were extracted via surgical excision using scissors and forceps for further analysis.

For hematoxylin and eosin (H&E) staining and immunohistochemistry (IHC) staining, the teratomas were fixed overnight at 4°C in 4% PFA, embedded in paraffin and subsequently processed for staining and analysis. For scRNA-sequencing, the teratomas were cut into small pieces on ice and digested at 37°C with collagenase IV supplemented with 1 U/mL DNase for 30 min. Single cells were re-suspended in 0.04% BSA for the 10x Genomics Chromium platform.

**Blastocyst chimera assay**

All mouse embryo microinjection and transplantation experiments were performed at Beijing Vitalstar Biotechnology Laboratory. EGFP- or mCherry-labelled TELSCs and TSCs were digested into single cells using TrypLE, washed twice with cold PBS and resuspended in culture medium. The cell suspension were incubated on ice for at least 30 min before microinjection.

Before microinjection, mouse embryos at 8-cell or blastocyst stage were obtained from the oviducts of superovulated ICR mice with M2 medium. Collected embryos were then transferred to M2 droplets and cultured under 5% CO_2_ at 37°C covered with mineral oil until further processing.

To create chimeric placentas, 10–15 TELSCs or TSCs were microinjected into each 8-cell or blastocyst stage ICR mouse embryo, respectively. After microinjection, embryos were recovered in KSOM drops for 1–2 h. Then, 10–15 chimeric embryos were transferred to a surrogate mouse and harvested at the E10.5–E13.5 developmental stage for analysis.

Conceptuses were dissected on ice in PBS/10% FBS, and the fetus, yolk sac and placenta were separated from the conceptuses with fine pointed forceps and imaged using a stereo fluorescence microscope (Leica, M205 FCA) to localize EGFP^+^ cells. After washing with PBS, tissues were then minced into approximately 1 mm fragments on ice using fine-pointed forceps. Placentas were digested at 37°C for 30 min with collagenase IV (Gibco, 17104019) supplemented with 1 U/mL DNase (Promega, M6101). PBS with 10% FBS was added to stop the reaction, and cells were centrifuged at 800 rpm for 5 min at 4°C. After resuspension in PBS/0.3% BSA, and the percentage of EGFP+ cells in the digested tissues was analyzed by flow cytometry.

**Flow cytometry**

ESCs and TELSCs were digested into single cells using 0.25% trypsin-EDTA or TrypLE and resuspended in 2% FBS (diluted with PBS (BI, 02—023—1ACS)). The cells were washed once with 4 mL of 2% FBS, resuspended in 100 μL of 2% FBS, and incubated on ice for 10 minutes. Then, fluorescence-conjugated primary antibodies (PE-CD40, BioLegend, 124605) were added at predetermined optimum concentrations and incubated on ice for 20 minutes in the dark. The samples were washed 3 times with at least 2 mL of 2% FBS by centrifugation at 800 rpm for 3 minutes. The cell pellet was resuspended in 0.5 mL of 2% FBS, and DAPI solution (Solarbio, C0060) was added to exclude dead cells if necessary. The cell suspension was then filtered through 40 μm cell strainers. Fluorescence-activated cell sorting (FACS) or flow cytometric analysis was performed. Data analysis was performed using FlowJo software. FACS-enriched cells were centrifuged and plated on gelatin/feeder/Matrigel-coated dishes for further culture.

**Western blotting**

ESCs and TELSCs were lysed in cold lysis buffer (20 mM Tris-HCl (pH 7.6), 137 mM NaCl, 1 mM EDTA, 1% Triton X-100, 1.5 mM MgCl_2_) supplemented with 1 mM PMSF (Beyotime, ST506) and then mixed with 4 × NuPAGE™ LDS sample buffer (Invitrogen, NP0007) before incubation at 98°C for 10 minutes. Then, the protein samples were loaded for SDS-PAGE and transferred to PVDF membranes (Millipore, IPVH07850) using a Bio-Rad transfer apparatus. The membrane was blocked with 5% milk (diluted in TBS buffer containing 0.1% Tween-20 (TBST) at room temperature for 1 hour, followed by incubation with primary antibody overnight at 4°C. After 3 washes with TBST, the membranes were incubated with appropriate HRP-conjugated secondary antibodies at room temperature for 1 hour with shaking. After three washes in TBST, the membrane was imaged with an Amersham Imager 600 (GE Healthcare).The following primary antibodies were used: anti-CDX2 (Abcam, ab76541), anti-EOMES (Abcam, ab216870), anti-OCT4 (Abcam, ab18976), and anti-β-Tubulin (Cell Signaling Technology, 2146).

**Derivation of trophoblast organoids from TELSCs**

After FACS, TELSCs were centrifuged and re-suspended in an appropriate volume of growth factor-reduced Matrigel (Corning, 354230) on ice to generate 30 μL Matrigel containing 0.5-1.0×10^4^ cells. Drops (30 µL) were plated into each well of a 24-well culture plate, maintained at 37°C for 15 minutes and overlaid with 800 µL trophoblast organoid medium (TOM). Cultures were maintained in 5% CO_2_ in a humidified incubator at 37°C. The medium was replaced every 2 days. Small organoid clusters became visible by approximately day 7 and were collected for downstream experiments.

TOM is composed of Advanced DMEM/F12 (GIBCO, 12634-010), 100 × N2 supplement (Thermo, 17502048), 50 × B27 supplement minus vitamin A (Thermo, 12587010), 1.25 mM N-Acetyl-L-cysteine (SIGMA, A9165), 2 mM GlutaMax, 50 ng/mL recombinant EGF (PeproTech, 315-09-100), 1.5 µM CHIR99021 (Selleck, S2924), 80 ng/mL recombinant R-spondin-1 (MCE, HY-P7114), 100 ng/mL recombinant FGF-2 (PeproTech, 450-33-50), 50 ng/mL recombinant HGF (PeproTech, 315-23-20), 500 nM A83-01 (Tocris, 2939), 2.5 µM prostaglandin E2 (SIGMA, P0409), and 2 µM Y-27632 (Selleck, S1049). The medium was stored at 4°C for up to 1 week.

**qRT-PCR**

TRIzol reagent (Invitrogen, 15596026) was used to isolate total RNA. Briefly, 1 mL TRIzol reagent was added to the collected cells, and after 5 minutes of incubation at room temperature, 0.2 mL chloroform was added for phase separation and incubated for 2 minutes. Then the samples were centrifuged for 20 minutes at 14,000 × rpm at 4°C. The upper aqueous phase was transferred to a new 1.5 mL tube and 0.5 mL isopropanol was added to precipitate the RNA. After mixing, the samples were incubated for 1 hour at -20°C, and then centrifuged for 30 minutes at 14,000 × rpm at 4°C. The supernatant was discarded and the RNA pellets were washed with 75% ethanol. Finally, the RNA pellets were air-dried and dissolved in RNase-free water. For mRNA qRT-PCR analysis, 500 μg RNA was converted into cDNA via reverse transcription using HiScript IIQRT SuperMix for qPCR (Vazyme, R223). Then, 2× Real-time PCR Mix (Mei5 Biotech, MF013) and an Applied Biosystems SteponePlus Real-Time PCR System (Thermo Fisher) were used to quantify the cDNA in duplicate or triplicate. Gene expression was normalized to Gapdh. The primers used are listed in **Table S8**.

**Immunofluorescence of cultured cell, organoids, and embryos**

ESCs and TELSCs were grown on gelatin/Matrigel-coated glass coverslips. The cells were fixed with fresh 4% paraformaldehyde (PFA)/PBS for 20 minutes at room temperature, washed three times with PBS, and permeabilized in 0.2% Triton X-100/PBS for 10 minutes at room temperature. The cells were blocked with 3% BSA/PBS and incubated with the primary antibody diluted in 3% BSA/PBS overnight at 4℃. Cells were then washed three times with PBS, incubated with secondary antibodies for one hour at room temperature, washed in PBS for three times, mounted with DAPI and imaged with a confocal microscope (Dragonfly High Speed Spinning Disk Confocal Microscope).

Organoids were grown in 35 mm confocal dishes (Cellvis, D35-20-1-N), fixed in 4% PFA for 30 minutes at room temperature, washed three times in PBS, permeabilized for 30 minutes in 0.5% Triton X-100/PBS, washed in PBS for three times and blocked in 5% BSA/PBS for 40 minutes at room temperature. Primary antibodies were incubated in 5% BSA/PBS with 0.05% Triton at 4℃ overnight. Organoids were washed three times for 15 minutes in PBS and incubated for 3 hours at room temperature in 5% BSA/PBS with secondary antibodies. Organoids were mounted with DAPI and imaged with a confocal microscope (Dragonfly High Speed Spinning Disk Confocal Microscope).

The following primary antibodies were used: anti-CDX2 (Abcam, ab76541)/(BioGenex, MU392A-UC), anti-EOMES (Abcam, ab216870), anti-ELF5 (Santa Cruz, sc-376737), anti-GATA3 (Abcam, ab199428), anti-HAND1 (Santa Cruz, sc-390376), anti-TPBPA (Abcam, ab104401), anti-STRA6 (R&D Systems, ABN1662), anti-KRT7 (Abcam, ab181598), anti-IGF1R (Abcam, ab182408), anti-PROLIFERIN (Santa Cruz, 271891), anti-OCT4 (Abcam, ab181557), anti-SOX2 (Abcam, ab137385), anti-β-Tubulin (Cell Signaling Technology, 2146), and anti-KIP2 (Abcam, ab133531).

**PolyA(+) RNA-seq**

For sample preparation, 1 μg of total RNA and a VAHTS Universal V8 RNA-seq Library Prep Kit (Vazyme, NR605) were used according to the manufacturer’s instructions. Library samples were subjected to the Illumina HiSeq X Ten sequencing system. For the analysis of RNA-seq data, Trimmomatic software was used to trim the adapters. HISAT2 software was used for alignment to the mm10 reference genome with default parameters. The expression count matrix was obtained by FeatureCounts. Downstream analysis was performed with R (version 3.6.2).

**Whole genome bisulfite sequencing (WGBS)**

In total, 100 ng genomic DNA was used as input and was bisulfite-converted using the EpiArt DNA Methylation Bisulfite Kit (Vazyme, EM101-01). Bisulfite-converted DNA was used to construct the library with the EpiArtTM DNA Methylation Library Kit V3 (Vazyme, NE103-C2) according to the manufacturer’s instructions. Library samples were subjected to an Illumina Nova-seq 6000 sequencing system.

**Bisulfite genomic sequencing (BSP)**

1 μg of genomic DNA from ESCs, TSLs and TOs were processed for bisulfite sequencing analysis using the Epitect Bisulfite Kit (QIANGEN, 59104) according to the manufacturer’s instructions. PCR reactions were performed for all genes analyzed (including Elf5, Oct4, and Nanog) using ZymoTaq PreMix (Zymo, E2004).

**CUT&Tag**

A total of 5×10^4^ viable cells from ESCs, TSLs and TOs were used for CUT&Tag experiments with the Hyperactive Universal CUT&Tag Assay Kit for Illumina (Vazyme, TD903) according to the manufacturer’s instructions. Library samples were subjected to the Illumina HiSeq X Ten sequencing system.

**ATAC-seq library preparation and sequencing**

ATAC-seq was performed using the TruePrep DNA Library Prep Kit V2 for Illumina (Vazyme, TD501). A total of 5×10^4^ viable cells from ESCs, TELSCs and TOs were digested and collected by centrifugation at 800 rpm for 5 minutes. The cell pellets were resuspended in cold lysis buffer (1M Tris-HCl, pH 7.4, 5 M NaCl, 1 M MgCl_2_, 0.1% NP40, 0.1% Tween-20 and 0.01% digitonin) and incubated on ice for 3 minutes. Then, the cell pellets were washed with 1 mL of wash buffer (1 M Tris-HCl, pH 7.4, 5 M NaCl, 1 M MgCl_2_ and 0.1% NP40) by inverting the tube three times. The cells were collected by centrifugation at 500 rcf for 10 minutes, and the cell pellets were resuspended in 50 mL transposition mix according to the manufacturer’s instructions. Next, the samples were subjected to an Illumina Nova-seq 6000 sequencing system.

**Single-cell RNA sequencing**

Mouse TOs were dissociated at day 9 as described previously (Karvas et al., 2022). In brief, organoids were removed from Matrigel in cell recovery solution at 4°C for 30 minutes. Organoids were collected and washed in ice-cold HBSS. Activated Papain solution was added and incubated at 37°C for 10 minutes. 10% FBS in DMEM was added to finish the digestion. 10 ml of DNase I (Promega, M6101) was added to each tube and incubated at 37°C for 5 minutes. Samples were centrifuged at 4000 RPM for 30 seconds. The cells were resuspended in PBS+0.04% BSA. Single-cell libraries were constructed using the 10× Single-cell 3ʹ Library & Gel Bead Kit v3.1 according to the manufacturer’s protocol.

**QUANTIFICATION AND STATISTICAL ANALYSIS**

**PolyA(+) RNA-seq data processing**

All raw reads were first trimmed by Trimmomatic (version 0.39) (Bolger et al., 2014) software to remove adapters and low-quality reads. Then, clean data were mapped to human genome (hg38) using HISAT2 (version 2.1.0) with default parameters. The count matrix of gene expression in each sample was generated by FeatureCounts (version 1.6.4) (Liao et al., 2014). After the genes were normalized to all mapped reads of each sample (CPM: count per million), gene expression changes were calculated based on the following formula: FC (fold change) = (CPMa+5)/(CPMb+5). We filtered significantly changed genes with different cutoffs, as indicated in the figure legends. Pheatmap R package was ultimately used to visualize the dynamic gene expression of samples. The published sequence fastq files were downloaded from different databases. RNA-seq of mouse embryos data (GSE98150), RNA-seq of mouse trophectoderm data (GSE163379), RNA-seq of mouse ectoplacental cone data (GSE65808), RNA-seq of mouse placentas data (GSE112755) and RNA-seq of TBLCs data (GSE168728) were obtained from GEO database. These data were also analyzed using the same methods described above.

**Single-cell RNA-seq data processing**

Single-cell transcriptome data were first applied to Cell Ranger (version 3.6.0) against hg38 genome reference to generate count matrix, which of the column was cell name and the row was gene. The downstream analysis was performed in Seurat (version 3.1.0) R package (Stuart et al., 2019). Then, we merged the single-cell data with published data (Jiang et al., 2023) directly. The cells from different samples can be detected in each lineage and mixed very well in each cluster indicating that the batch effect did not affect the cell clustering. The read count data were normalized by NormalizeData with “LogNormalize” method. We chose 1-30 PCs after RunPCA function to perform RunUMAP function and cell clusters were investigated by a shared nearest neighbor (SNN) modularity optimization with FindClusters function with parameter “resolution = 0.4”. Next, we used function “FindAllMarkers” to identify DEGs. Genes with “min.pct = 0.5, logfc.threshold = 0.25” were considered significantly different.

**ATAC-seq data processing**

ATAC-seq data were firstly trimmed by Trimmomatic software (version 0.39). Bowtie2 (version 2.4.4) (Langmead et al., 2019) was used to align the clean reads to mm10 reference genome “-X 1000 –mm -local”. Picard was used to remove duplicates and we only remained the high-quality reads. Then, after calling the peaks by MACS2, we used the merge and multicov commands in bedtools to extract the count of the peaks in all the samples and bdgdiff function in MACS2 was used to normalize the count matrix and calculate the differential peaks with parameters “-g 100 -l 50 -C 2”. ATAC-seq data of mTBLC were downloaded from GEO database (GSE168728), and analyzed using the same method.

**ChIP-seq data processing**

ChIP-seq data were firstly trimmed by Trimmomatic software. Bowtie2 (version 2.4.4) (Langmead et al., 2019) was used to align the clean reads to mm10 reference genome with parameters “--end-to-end --very-sensitive --no-mixed --no-discordant --phred33 -I 10 -X 700”. We used MACS2 (version 2.1.4) (Zhang et al., 2008) to call peaks and differential peaks were detected by macs2 bdgdiff command.

**Principal component and unsupervised hierarchical clustering analysis**

We used PCA function in FactoMineR (version 1.42) and hclust function to perform PCA and clustering analysis, respectively. Due to the batch effects in different projects, Combat function in sva (version 3.34.0) (Leek et al., 2012) R package was used to remove the batch effect between RNA-seq data obtained from different studies.

**Gene set variation analysis (GSVA)**

Gene set variation analysis was carried out using the GSVA (version 1.32.0) R package.

**Gene set enrichment analysis (GSEA)**

GSEA was performed using row counts with GSEA software (version 4.1.0). The enrichment score was calculated by GSEA function in cluster Profiler R package.

**Gene ontology annotation**

ClusterProfiler (version 3.14.3) R package was used for Gene Ontology (GO) term analysis. GO terms with p-value < 0.05 were defined as significant process.

**Pseudotime trajectory analysis**

Pseudotime trajectories were constructed using Monocle2 (2.22.0). Only genes with an average expression greater than 3 were retained for the analysis. The “FindVariableFeatures” function was used to identify highly variable genes for ordering cells. The “plot_cell_trajectory” function was used to visualize results annotated with cell type information.

**Alignment track visualization**

The visualization of different tracks was performed using IGV.

# Supplemental Information

Table S1. Lists of differentially expressed genes in TBLCs, TELCs, TELSCs, TESC (Seong et al., 2022) and TSC, related to Figure 1

Table S2. Lists of differentially expressed genes in TE3.5-ExE6.5 cells used in GSVA, related to Figure 1K

Table S3. Lists of differentially expressed genes in MEF-, TSC- and TELSC derived teratoma, related to Figure S4E

Table S4. Lists of differentially expressed genes in in MEF-, TSC- and TELSC derived teratoma used in GSVA, related to Figure 4F

Table S5. Marker genes for each defined trophoblast cell type (ExE-like, LaTP, SpT, SynTI, SyntII, GlyT, S-TGC, P-TGC), related to Figure 6

Table S6. Lists of differentially expressed genes in TELSC, TELSC-derived organoids at different time points and organoids reported by Mao et al.,, related to Figure 7C

Table S7. Summary of all sequencing data in this study

Table S8. Lists of primers and sgRNA sequences used in this study

References

Akalin, A., Kormaksson, M., Li, S., Garrett-Bakelman, F.E., Figueroa, M.E., Melnick, A., and Mason, C.E. (2012). methylKit: a comprehensive R package for the analysis of genome-wide DNA methylation profiles. Genome Biology 13.

Andrews, S., Krueger, C., Mellado-Lopez, M., Hemberger, M., Dean, W., Perez-Garcia, V., and Hanna, C.W. (2023). Mechanisms and function of de novo DNA methylation in placental development reveals an essential role for DNMT3B. Nat Commun 14, 371.

Bolger, A.M., Lohse, M., and Usadel, B. (2014). Trimmomatic: a flexible trimmer for Illumina sequence data. Bioinformatics 30, 2114-2120.

Cheng, S., Pei, Y., He, L., Peng, G., Reinius, B., Tam, P.P.L., Jing, N., and Deng, Q. (2019). Single-Cell RNA-Seq Reveals Cellular Heterogeneity of Pluripotency Transition and X Chromosome Dynamics during Early Mouse Development. Cell Reports 26.

Chu, A., Casero, D., Thamotharan, S., Wadehra, M., Cosi, A., and Devaskar, S.U. (2019). The Placental Transcriptome in Late Gestational Hypoxia Resulting in Murine Intrauterine Growth Restriction Parallels Increased Risk of Adult Cardiometabolic Disease. Sci Rep 9, 1243.

Durinck, S., Moreau, Y., Kasprzyk, A., Davis, S., De Moor, B., Brazma, A., and Huber, W. (2005). BioMart and Bioconductor: a powerful link between biological databases and microarray data analysis. Bioinformatics 21, 3439-3440.

Hänzelmann, S., Castelo, R., and Guinney, J. (2013). GSVA: gene set variation analysis for microarray and RNA-Seq data. BMC Bioinformatics 14.

Hon, G.C., Rajagopal, N., Shen, Y., McCleary, D.F., Yue, F., Dang, M.D., and Ren, B. (2013). Epigenetic memory at embryonic enhancers identified in DNA methylation maps from adult mouse tissues. Nature Genetics 45, 1198-1206.

Jiang, X., Wang, Y., Xiao, Z., Yan, L., Guo, S., Wang, Y., Wu, H., Zhao, X., Lu, X., Wang, H.*, et al.* (2023). A differentiation roadmap of murine placentation at single-cell resolution. Cell Discov 9, 30.

Kim, D., Paggi, J.M., Park, C., Bennett, C., and Salzberg, S.L. (2019). Graph-based genome alignment and genotyping with HISAT2 and HISAT-genotype. Nature Biotechnology 37, 907-915.

Koh, P.W., Sinha, R., Barkal, A.A., Morganti, R.M., Chen, A., Weissman, I.L., Ang, L.T., Kundaje, A., and Loh, K.M. (2016). An atlas of transcriptional, chromatin accessibility, and surface marker changes in human mesoderm development. Scientific Data 3.

Krueger, F., and Andrews, S.R. (2016). SNPsplit: Allele-specific splitting of alignments between genomes with known SNP genotypes. F1000Research 5.

Langmead, B., Wilks, C., Antonescu, V., Charles, R., and Hancock, J. (2019). Scaling read aligners to hundreds of threads on general-purpose processors. Bioinformatics 35, 421-432.

Leek, J.T., Johnson, W.E., Parker, H.S., Jaffe, A.E., and Storey, J.D. (2012). The sva package for removing batch effects and other unwanted variation in high-throughput experiments. Bioinformatics 28, 882-883.

Li, H., Handsaker, B., Wysoker, A., Fennell, T., Ruan, J., Homer, N., Marth, G., Abecasis, G., and Durbin, R. (2009). The Sequence Alignment/Map format and SAMtools. Bioinformatics 25, 2078-2079.

Liao, Y., Smyth, G.K., and Shi, W. (2014). featureCounts: an efficient general purpose program for assigning sequence reads to genomic features. Bioinformatics 30, 923-930.

Liu, X., Wang, C., Liu, W., Li, J., Li, C., Kou, X., Chen, J., Zhao, Y., Gao, H., Wang, H.*, et al.* (2016). Distinct features of H3K4me3 and H3K27me3 chromatin domains in pre-implantation embryos. Nature 537, 558-562.

Mao, Q., Ye, Q., Xu, Y., Jiang, J., Fan, Y., Zhuang, L., Liu, G., Wang, T., Zhang, Z., Feng, T.*, et al.* (2023). Murine trophoblast organoids as a model for trophoblast development and CRISPR-Cas9 screening. Dev Cell 58, 2992-3008 e2997.

Nowotschin, S., Setty, M., Kuo, Y.Y., Liu, V., Garg, V., Sharma, R., Simon, C.S., Saiz, N., Gardner, R., Boutet, S.C.*, et al.* (2019). The emergent landscape of the mouse gut endoderm at single-cell resolution. Nature 569, 361-367.

Ritchie, M.E., Phipson, B., Wu, D., Hu, Y., Law, C.W., Shi, W., and Smyth, G.K. (2015). limma powers differential expression analyses for RNA-sequencing and microarray studies. Nucleic Acids Research 43, e47-e47.

Seong, J., Frias-Aldeguer, J., Holzmann, V., Kagawa, H., Sestini, G., Heidari Khoei, H., Scholte Op Reimer, Y., Kip, M., Pradhan, S.J., Verwegen, L.*, et al.* (2022). Epiblast inducers capture mouse trophectoderm stem cells in vitro and pattern blastoids for implantation in utero. Cell Stem Cell 29, 1102-1118 e1108.

Shen, H., Yang, M., Li, S., Zhang, J., Peng, B., Wang, C., Chang, Z., Ong, J., and Du, P. (2021). Mouse totipotent stem cells captured and maintained through spliceosomal repression. Cell 184, 2843-2859 e2820.

Stuart, T., Butler, A., Hoffman, P., Hafemeister, C., Papalexi, E., Mauck, W.M., Hao, Y., Stoeckius, M., Smibert, P., and Satija, R. (2019). Comprehensive Integration of Single-Cell Data. Cell 177, 1888-1902.e1821.

Sun, J., Zheng, W., Liu, W., Kou, X., Zhao, Y., Liang, Z., Wang, L., Zhang, Z., Xiao, J., Gao, R.*, et al.* (2021). Differential Transcriptomes and Methylomes of Trophoblast Stem Cells From Naturally-Fertilized and Somatic Cell Nuclear-Transferred Embryos. Front Cell Dev Biol 9, 664178.

Tuteja, G., Chung, T., and Bejerano, G. (2016). Changes in the enhancer landscape during early placental development uncover a trophoblast invasion gene-enhancer network. Placenta 37, 45-55.

Wang, C., Liu, X., Gao, Y., Yang, L., Li, C., Liu, W., Chen, C., Kou, X., Zhao, Y., Chen, J.*, et al.* (2018). Reprogramming of H3K9me3-dependent heterochromatin during mammalian embryo development. Nat Cell Biol 20, 620-631.

Weigert, R., Hetzel, S., Bailly, N., Haggerty, C., Ilik, I.A., Yung, P.Y.K., Navarro, C., Bolondi, A., Kumar, A.S., Anania, C.*, et al.* (2023). Dynamic antagonism between key repressive pathways maintains the placental epigenome. Nature Cell Biology 25, 579-591.

Wu, H., Wang, C., and Wu, Z. (2013). A new shrinkage estimator for dispersion improves differential expression detection in RNA-seq data. Biostatistics 14, 232-243.

Yu, G., Wang, L.-G., Han, Y., and He, Q.-Y. (2012). clusterProfiler: an R Package for Comparing Biological Themes Among Gene Clusters. OMICS: A Journal of Integrative Biology 16, 284-287.

Zhang, Y., Liu, T., Meyer, C.A., Eeckhoute, J., Johnson, D.S., Bernstein, B.E., Nusbaum, C., Myers, R.M., Brown, M., Li, W.*, et al.* (2008). Model-based Analysis of ChIP-Seq (MACS). Genome Biology 9.
